# The effectiveness of clinical guideline implementation strategies in oncology—a systematic review

**DOI:** 10.1186/s12913-023-09189-x

**Published:** 2023-04-06

**Authors:** Ana-Mihaela Bora, Vanessa Piechotta, Nina Kreuzberger, Ina Monsef, Andreas Wender, Markus Follmann, Monika Nothacker, Nicole Skoetz

**Affiliations:** 1 Evidence-Based Medicine, Department I of Internal Medicine, Center for Integrated Oncology Aachen Bonn Cologne Duesseldorf, Faculty of Medicine and University Hospital Cologne, University of Cologne, Cologne, Germany; 2 German Cancer Society, Berlin, Germany; 3 Institute for Medical Knowledge Management, Association of the Scientific Medical Societies in Germany, C/O Faculty of Medicine, Philipps University Marburg, Marburg, Germany

**Keywords:** Clinical practice guideline, Implementation, Oncology, Patient-relevant outcomes, Healthcare professionals, Guideline adherence, Behaviour

## Abstract

**Importance:**

Guideline recommendations do not necessarily translate into changes in clinical practice behaviour or better patient outcomes.

**Objective:**

This systematic review aims to identify recent clinical guideline implementation strategies in oncology and to determine their effect primarily on patient-relevant outcomes and secondarily on healthcare professionals' adherence.

**Methods:**

A systematic search of five electronic databases (PubMed, Web of Science, GIN, CENTRAL, CINAHL) was conducted on 16 december 2022. Randomized controlled trials (RCTs) and non-randomized studies of interventions (NRSIs) assessing the effectiveness of guideline implementation strategies on patient-relevant outcomes (overall survival, quality of life, adverse events) and healthcare professionals' adherence outcomes (screening, referral, prescribing, attitudes, knowledge) in the oncological setting were targeted. The Cochrane risk-of-bias tool and the ROBINS-I tool were used for assessing the risk of bias. Certainty in the evidence was evaluated according to GRADE recommendations. This review was prospectively registered in the International Prospective Register of Systematic Reviews (PROSPERO) with the identification number CRD42021268593.

**Findings:**

Of 1326 records identified, nine studies, five cluster RCTs and four controlled before-and after studies, were included in the narrative synthesis. All nine studies assess the effect of multi-component interventions in 3577 cancer patients and more than 450 oncologists, nurses and medical staff.

***Patient-level*:**

Educational meetings combined with materials, opinion leaders, audit and feedback, a tailored intervention or academic detailing may have little to no effect on overall survival, quality of life and adverse events of cancer patients compared to no intervention, however, the evidence is either uncertain or very uncertain.

***Provider-level*:**

Multi-component interventions may increase or slightly increase guideline adherence regarding screening, referral and prescribing behaviour of healthcare professionals according to guidelines, but the certainty in evidence is low. The interventions may have little to no effect on attitudes and knowledge of healthcare professionals, still, the evidence is very uncertain.

**Conclusions and relevance:**

Knowledge and skill accumulation through team-oriented or online educational training and dissemination of materials embedded in multi-component interventions seem to be the most frequently researched guideline implementation strategies in oncology recently. This systematic review provides an overview of recent guideline implementation strategies in oncology, encourages future implementation research in this area and informs policymakers and professional organisations on the development and adoption of implementation strategies.

**Supplementary Information:**

The online version contains supplementary material available at 10.1186/s12913-023-09189-x.

## Key points

**Question:** What are the most effective clinical guideline implementation strategies in oncology?

**Findings:** The nine included studies assessed multi-component guideline implementation interventions compared to no intervention. Educational meetings combined with materials, opinion leaders, audit and feedback, a tailored intervention or academic detailing may have little to no effect on overall survival, quality of life and adverse events of cancer patients compared to no intervention, however, the evidence is either uncertain or very uncertain. Multi-component interventions may increase or slightly increase guideline adherence regarding screening, referral and prescribing behaviour of healthcare professionals according to guidelines, but the certainty in evidence is low. The interventions may have little to no effect on attitudes and knowledge of healthcare professionals, still, the evidence is very uncertain.

**Meaning:** This systematic review gives an overview of recent strategies used for guideline implementation in oncology in order to inform policymakers and professional organisations on the development and adoption of implementation strategies.

## Introduction

Clinical practice guidelines (CPGs) are a powerful tool of evidence-based medicine, designed to mitigate the gap between clinical research and current practice [1, 2]. It was shown that non-adherence to guidelines may lead to unnecessary diagnostics and suboptimal treatment [3–5]. On the contrary, a systematic review concluded that adherence to breast cancer guidelines was associated with increased overall survival and disease-free survival [6]. The implementation of CPGs in oncology is considered to be very complex and therefore challenging due to the heterogeneity of cancer types, high number of CPGs of different methodologies, inconsistent use of guideline-based quality indicators, complexity of therapeutic decisions, and the various influences of multiple interconnected clinical specialties involved in this setting [7, 8]. This may lead to inconsistencies and patient and practitioner confusion due to information overload [9, 10]. Also, the heterogeneity in structure, target groups and endpoints addressed in guidelines may be a challenge for implementation, as discovered by comparing nine oncological CPGs of well-known organisations on advanced breast, lung, and colon cancer [11]. Due to these barriers, recommendations may not be adequately applied in practice and patients may not benefit from evidence-based research. The use of CPGs in practice is reported as being unpredictable and slow [12]. It was estimated that approximately 30–50% of patients receive treatment that is not evidence-based, and 20–25% receive unnecessary or even potentially harmful treatments [13]. For example, a study concluded that guideline-discordant imaging appears to be common as almost half of men with low-risk localised prostate cancer receive unnecessary imaging while there is underuse of imaging among men with a high-risk disease in the USA [14]. Furthermore, it was shown that nurses' failure to routinely screen and implement appropriate cancer pain management has an adverse impact on health-related quality of life [15]. Moreover, another study showed that urgent referral guideline recommendations were not followed for the majority of patients with common possible cancer features in the UK [4]. Consequently, the development of high methodological CPGs alone does not automatically result in their use. In order to improve patient outcomes and decrease variations in the current oncological practice, it is important to identify and assess optimal strategies for the implementation of CPGs [6, 7, 16].

There are various implementation strategies that have been tested over the years. These strategies can be used alone as single-component strategies or in combination as multi-component interventions to facilitate the use of CPGs in clinical practice. The dissemination of printed educational materials has been considered as accessible, convenient to use, and potentially cost-effective intervention across healthcare settings [17]. It was shown that used alone and compared to no intervention, it may have a small beneficial effect on professional practice outcomes. The effect of opinion leaders was examined in a recent Cochrane review which concluded that used alone or in combination with other implementation strategies, it probably improves the compliance with evidence-based practice of professionals [18]. Further, reminders (manually and computer-generated) were shown to probably improve the quality of care compared to usual care or other co-interventions [19, 20]. Moreover, it was shown that audit and feedback lead to small but potentially important improvements in professional practice. The effectiveness of guideline implementation strategies seems to depend on how the feedback is provided and on the baseline performance of professionals [21].

The systematic review of Grimshaw 2004 found that 73% of the included studies examined multi-component interventions, and the most effective single strategies were reminders, dissemination of educational materials, and audit and feedback [22]. In the hospital setting of emergency departments, reminders alone or educational interventions combined with audit and feedback were likely to be effective in improving guideline adherence [23]. In the care of chronic diseases at the primary level setting, passively receiving educational materials was least effective compared to educational meetings implying the active involvement of professionals [24]. Multi-component interventions were slightly more effective compared to single interventions [24, 25]. Still, in all these reviews, although assessing change in health care provider behaviours, it was rather uncertain whether the interventions really lead to improved patient outcomes.

One review concluded that reminders and feedback as a single intervention, and group education and organisational strategies used as part of a multi-component intervention, corresponded with positive changes on professionals’ behaviour and patient outcomes in the oncological setting [26]. Still, this review relies mostly on studies published more than ten years ago. Moreover, the research findings from Grimshaw [22] and Hakkennes and Dodd [27] serve as a foundation for understanding CPG implementation strategies among professionals, yet they rely on papers published almost 20 years ago and are not specific to oncology. Other more recent reviews assess the effectiveness of implementation strategies, however, do not particularly focus on oncology [24, 25].

Despite the current interest in CPGs and innovative methods to promote knowledge transfer into practice, a surprisingly high uncertainty about the effectiveness of guideline implementation strategies in oncology remains. This systematic review aims to fill the gap regarding the synthesis of the effectiveness of recent guideline implementation strategies on patient-relevant outcomes and guideline adherence of healthcare professionals in the oncological settings.

## Methods

This systematic review was performed according to the recommendations of the Cochrane Handbook for Systematic Reviews of Interventions and the Preferred Reporting Items for Systematic Reviews and Meta-Analyses (PRISMA) statement [28, 29]. The PICO (*P*opulation, *I*ntervention, *C*omparison, *O*utcome) framework was used to guide the eligibility criteria of this review (Table 1). To identify most recent studies, a comprehensive electronic literature search for studies as of year 2011 was performed. The following electronic databases were searched on 16 December 2022: PubMed, Web of Science, GIN, CENTRAL and CINAHL. With the assistance of an experienced information specialist (IM), these search strategies were optimised. Search filters were used to identify papers with study designs of interest (e.g. filter for RCTs). The search strategies included keywords such as *clinical practice guideline, implementation, survival, adherence, behaviour, health professionals, patients, oncology*. The full search strategies can be found in Additional file 1 in the Supplement of this review.

**Table 1:** PICO framework

P	I	C	O
**Population**	**Intervention**	**Comparison**	**Outcome**
Healthcare professionals (physicians, nurses, etc.)	CPG implementation of single or multi-component interventions^a^ in the oncological setting or any clinical setting related to oncology	No intervention (usual care), another single-component intervention or multi-component intervention	**Primary (patient-level)**: Overall survival, Quality of Life, Adverse event(s) **Secondary (provider-level)**: Guideline adherence outcomes^b^

*CPG* Clinical practice guideline

^a^single intervention = single-component or single strategy interventions (e.g. reminders only), multi-component intervention = intervention with two or more components or strategies (e.g. reminders combined with audit and feedback)

^b^defined as screening rate, referral, prescribing behaviour, knowledge, attitudes of healthcare professionals

Screening and selection of studies were performed independently by two reviewers (AB, VP, NK). Reference lists of all eligible studies and relevant systematic reviews were hand searched by one author (AB) for additional eligible studies. Only prospectively registered controlled studies (e.g. (cluster) randomized controlled trials, controlled pre-post trial designs) were included in this review. The risk of bias of each included study was independently assessed by pairs of two reviewers (AB, VP, AW, NK). Disagreements were resolved by discussion and consensus and a third reviewer (NS) was involved when consensus was not reached. The Cochrane risk of bias tool for RCTs quality assessment and ROBINS-tool for non-randomized studies were used [30, 31]. Overall, a study was judged to have a high risk of bias if at least one bias domain was judged to be at high risk. Moreover, the certainty in the evidence was rated for all outcomes using the GRADE tool [32] and recommendations [33]. It was judged by one reviewer (AB) and checked by two other reviewers (NK, NS). The assessment was separated according to the study design.

## Results

**Fig. 1:**
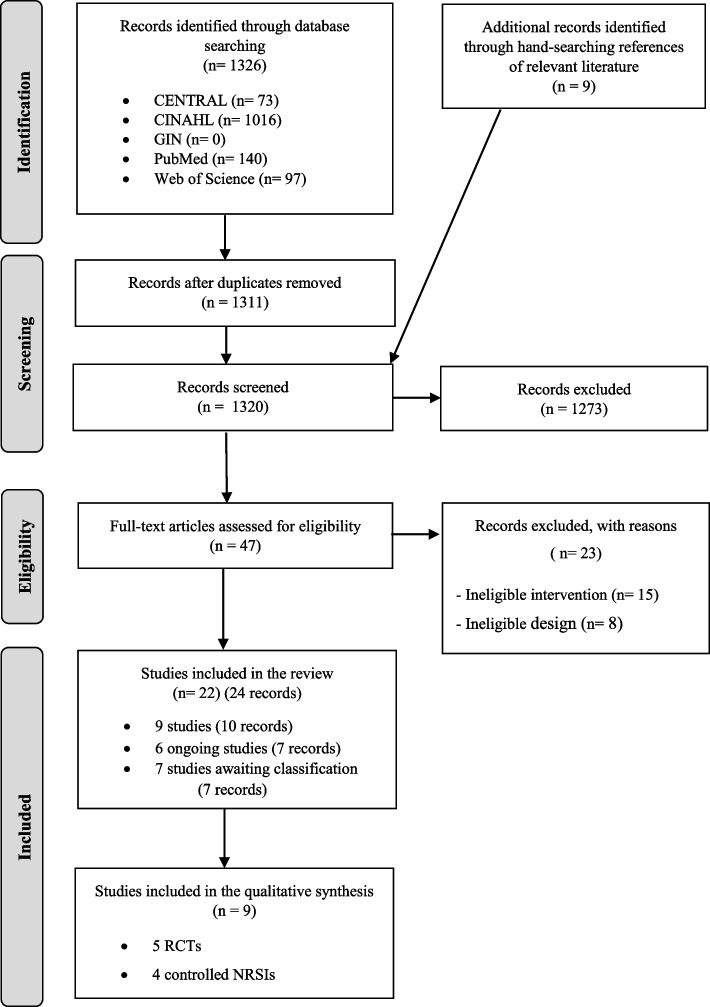
PRISMA flowchart. RCT = randomized controlled trial; NRSIs = non-randomized studies of interventions

A total of 1326 records were identified through electronic database searching. After identifying nine additional records through hand-searching references and removing fifteen duplicate records, a total of 1320 records were included for the title and abstract screening. The detailed study selection process is described in the PRISMA flow-diagram (Fig. 1). Furthermore, the list of excluded studies at full-text screening stage can be found in the Supplement (Additional file 2). A total of nine studies published between 2017 and 2022, five cluster RCTs [34–38] and four controlled NRSIs with before-and after study design [39–42], were included in the synthesis. Further, six randomized ongoing trials were identified [43–48] and seven studies were categorised as awaiting classification due to no published results [49–51] and conference abstracts with insufficient information [52–54] or inaccessible full text [55].

The nine included studies assessed multi-component guideline implementation interventions compared to no intervention in 3577 cancer patients and more than 450 oncologists, nurses and medical staff [34–42]. The most frequently used strategies, which were applied in all nine interventions, were educational meetings and educational materials (Table 2). Population characteristics of the included studies, a detailed description of the interventions, outcomes and characteristics of ongoing and awaiting assessment studies can be found in the Supplement (Additional files 3, 4,5, 6, 7)*.*

**Table 2:** Study characteristics, interventions and comparators

Study (Reference)	Trial identifier	Country	Study design	Study period	Setting	Clinical Practice Guideline(s)	Intervention^a^	Comparator
**Brown 2018** [38]	ACTRN12611001251910	Australia	stepped-wedge cluster RCT (9 clusters)	13.12.2013 - 27.08.2014	inpatient	Australian Guideline for the Management of Prostate Cancer (2010), American Urological Association Guideline (2013)	Multi-component (opinion leaders, educational meetings and materials, audit and feedback, tailored intervention)	No intervention
**Gilbert 2021** [37]	NCT0208452	France	stepped-wedge cluster RCT (5 clusters)	10.2013 - 12.2016	inpatient	ESPEN Guidelines on Nutrition in Cancer Patients (2017)	Multi-component (educational meetings and materials, academic detailing)	No intervention
**Lovell 2022** [34]	ACTRN12615000064505	Australia	stepped-wedge cluster RCT (6 clusters)	08.2015 - 05.2019	outpatient	Cancer Council Australia Cancer pain management in adults (2020)	Multi-component (opinion leaders, educational meetings and materials, audit and feedback)	No intervention
**McCarter 2018** [35]	ACTRN12613000320752	Australia	stepped-wedge cluster RCT (5 clusters)	01.07.2013 - 27.03.2015	inpatient	National Comprehensive Cancer Network (NCCN)- Guidelines in Oncology, Head and Neck Cancers (2017)	Multi-component (opinion leaders, educational meetings and materials, audit and feedback, academic detailing)	No intervention
**Mohile 2021** [36]	NCT02054741	USA	Cluster RCT	29.07.2014 - 13.03.2019	inpatient	ASCO Guideline for Geriatric Oncology (2018)	Multi-component (educational meetings and materials)	No intervention
**Bonkowski 2018** [40]	NR	USA	Controlled before-and after study	NR	inpatient	Guidelines on the Management of Postoperative Pain (2016)	Multi-component (educational meetings, educational materials, tailored intervention)	No intervention
**Cowperthwaite 2019** [42]	NR	USA	Controlled before and after study	07.07.2017 – 30.11.2017	inpatient	National Comprehensive Cancer Network (NCCN)- CPG in Oncology, Adult Cancer Pain (2018)	Multi-component (educational meetings, educational materials, patient-mediated intervention)	No intervention
**Knoerl 2021** [41]	NCT03514680	USA	Controlled before-and after study	05.2018 – 11.2019	outpatient	American Society of Clinical Oncology (ASCO)-CPG for prevention and management of CIPN in survivors of adult cancers (2020)	Multi-component (educational meetings, educational materials)	No intervention
**Phillips 2017** [39]	NR	Australia	Controlled before-and after study	NR (41 weeks)	inpatient	Australian Adult Cancer Pain Management Working Group- Cancer pain management in adults (2013)	Multi-component (educational meetings, educational materials, audit and feedback)	No intervention

*NR* Not reported, *CIPN* Chemotherapy-induced peripheral neuropathy, *RCT* Randomized controlled trial

^a^Classification according to EPOC taxonomy [56]; tailored intervention = based on an assessment of barriers to change, for example through interviews or surveys; patient-mediated intervention = any intervention aimed at changing the performance of healthcare professionals through interactions with patients, or information provided by or to patients

### Risk of bias in randomized studies

**Fig. 2:**
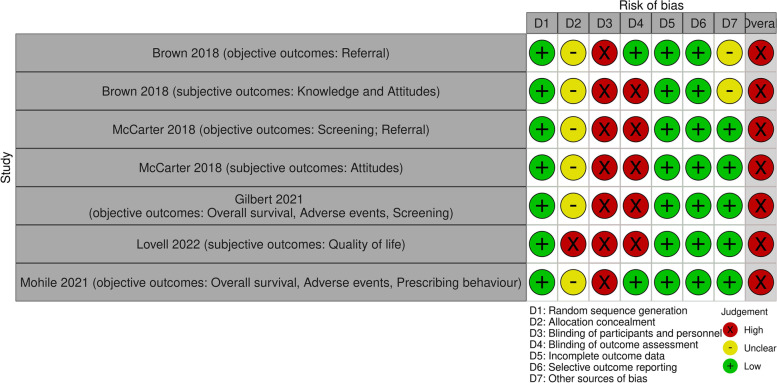
Risk of bias in randomized controlled trials

Overall, the risk of bias was rated as high for all five cluster RCTs [34–38] due to the lack of blinding of outcome assessors and participants, which affected both objective and subjective outcomes (Fig. 2). Additionally, the detailed risk of bias judgement table and the risk of bias summary plot are listed in Additional files 8 and 10 in the Supplement.

### Risk of bias in non-randomized studies

**Fig. 3:**
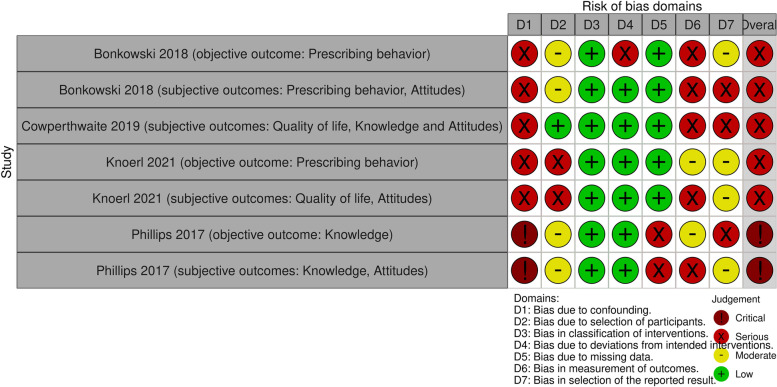
Risk of bias in non-randomized controlled trials

For both objective and subjective outcomes, the overall risk of bias was judged to be serious in three studies (studies have some important problems) [40–42] and critical in one study (the study is too problematic to provide any useful evidence and should not be included in synthesis) [39] (Fig. 3). This due to the lack of comparability between groups and lack of control for confounders, bias in the measurement and the reporting of outcomes. Additionally, the detailed risk of bias judgement table and the risk of bias summary plot are listed Additional files 9 and 10 in the Supplement.

### Effects on primary (patient-level) and secondary (provider-level) outcomes

The effects on primary and secondary outcomes of this review are summarised in Additional file 11 in the outcome effects Tables 1, 2, 3, 4, 5, 6, 7, 8 in the *Supplement* of this review.

#### Patient-level outcomes

### Overall survival

Two cluster RCTs reported overall survival in 865 cancer patients [36, 37]. Both studies suggested little to no difference in effects (HR 1.05, 95% CI: 0.85 to 1.29, *p =* 0.68; RR 0.946, 95% CI: 0.895 to 1.228, *p =* 0.813; eTable 1) [36, 37]. Overall, the effect of the implementation interventions may have little to no effect on overall survival, still, this is uncertain due to the serious risk of bias and imprecision of outcome measurement.

### Quality of life and patient-reported outcomes

One cluster RCT reported pain scores for 544 cancer patients [34]. The intervention combining opinion leaders with educational meetings, materials and audit and feedback may have little to no effect on pain scores and on total quality of life QLQ-C15-PAL scores measured at different follow-up times (eTable 2). The certainty in the evidence is low due to serious limitations in the study design and imprecision.

Two NRSIs reported quality of life as different symptoms measured with different scales in 472 patients [41, 42]. The time points reported in both studies were not clearly described. Measured on a scale from 0 (no pain) to 10 (worst pain), the results of Cowperthwaite 2019 suggested little to no difference between intervention and comparator at T1 (MD 0.090, 95% CI: -0.6131 to 0.7931, *p =* 0.8013; eTable 2) and a small effect at T2 (MD 0.210, 95% CI: -0.3477 to 0.7677, *p =* 0.4594; eTable 2) on pain intensity [42]. Still, the evidence is very uncertain. The results of Knoerl 2019 (eTable 2) suggested effects in favour of the intervention on CIPN sensory (at T1, T2, T3) and motor severity (at T1, T3) on a Lickert Scale from 1 to 4 [41], however, the certainty in the evidence is very uncertain. Overall, the effect of the implementation interventions compared to no intervention resulting from non-randomized studies on quality of life is very uncertain due to the very serious risk of bias, imprecision, and serious indirectness of outcome measurement.

### Adverse events

Two cluster RCTs reported adverse events in 865 cancer patients [36, 37]. Gilbert 2021 suggested an effect in favour of the comparator regarding the proportion of patients having at least one adverse event or one postsurgical complication (eTable 3), still, the evidence is very uncertain [37]. The results of Mohile 2021 suggested an effect in favour of the intervention at 3 months in terms of 3–5 grade adverse events (adjusted RR 0.74, 95% CI: 0.64 to 0.86, *p =* 0.001; eTable 3) [36]. Overall, the effect of the implementation interventions may have little to no effect on adverse events, still, the evidence is very uncertain due to the serious risk of bias, imprecision and inconsistency in outcome results.

#### Provider-level outcomes

### Screening

Two cluster RCTs reported screening for 454 cancer patients [35, 37]. Both studies combined educational meetings with educational materials, academic detailing. One study additionally combined opinion leaders with audit and feedback. Both suggested an effect in favour of the intervention (OR 348.82, 95% CI 69.31 to 1755.62, *p <* 0.0001; RR = 5.29, 95% CI 3.03 to 9.23, *p <* 0.0001; eTable 4). Overall, the multicomponent interventions implemented in these studies may increase adherence to guidelines regarding screening rates, still, the certainty of the evidence is low due to serious limitations in the study design and imprecision of results.

### Referral

Two cluster RCTs reported referrals for 1219 patients [35, 38]. The results of Brown 2018 suggested little to no difference between the intervention (educational meetings, materials, audit and feedback, opinion leaders, tailored intervention) and no intervention on referrals (RR 1.0474, 95% CI: 0.8631 to 1.2711, *p =* 0.6389; eTable 5) [38]. The results of McCarter 2018 suggested an effect in favour of almost the same intervention (used academic detailing instead of a tailored intervention) on referral rates (OR 37.70, 95% CI: 0.93 to 1530, *p =* 0.0537; eTable 5) [35]. Overall, the multicomponent interventions implemented in these studies may increase referrals slightly according to guidelines, still, the certainty of the evidence is low due to serious limitations in the study design and serious imprecision.

### Prescribing behaviour

One cluster RCT reported this outcome for 718 cancer patients [36]. Combining educational materials and meetings for guideline implementation may increase adherence to guidelines slightly regarding prescribing behaviour compared to no intervention (eTable 6). However, the certainty in the evidence is low due to serious limitations in the study design and serious imprecision.

Two NRSIs reported prescribing behaviour [40, 41]. The results of Bonkowski 2018 suggested effects in favour of the intervention on narcotic administration of one and three doses. Further, the evidence suggested little effect in one item (MD -0.440, 95% CI: -0.0867 to 0.7933, *p =* 0.0157; eTable 6), and no difference in another item (MD 0.000, 95% CI: -0.3354 to 0.3354, *p =* 1.000; eTable 6) [40]. Knoerl 2021 suggested an effect in favour of the intervention on the frequency of appropriate mild CIPN management (OR = 2.5278, 95% CI: 0.8356 to 7.6471, *p =* 0.1006; eTable 6), whereas the frequency for appropriate moderate-severe CIPN management (OR = 0.8571, 95% CI: 0.2463 to 2.9827, *p =* 0.8086; eTable 6) was lower after the intervention [41]. Overall, the interventions may have little to no effect on this outcome, but the evidence from non-randomized studies is very uncertain due to very serious limitations in the study design and serious imprecision.

### Attitudes

Six included studies reported this outcome using different measurements (eTable 11) [35, 38–42]. One RCT and one NRSI reported attitudes combined with knowledge [38, 42]. The other RCT reported that the majority of dieticians indicate that the implementation intervention was helpful or very helpful [35]. In one NRSI it was narratively reported that nurses were highly satisfied with the intervention [40]. Knoerl 2021 reported acceptability and feasibility scores only for the intervention group [41]. The results of Phillips 2017 suggested lower self-perceived knowledge directly after the intervention (MD 1.9, 95% CI: 0.5 to 3.4, *p =* 0.012; eTable 7) and an effect in favour of the intervention at ten weeks after (MD -1.4, 95% CI: -1.9 to -1.0, *p <* 0.001; eTable 7) [39]. Overall, the interventions may have little to no effect on attitudes, but the evidence is very uncertain due to serious (for RCTs) and very serious (for NRSIs) limitations in the study design, serious indirectness and very serious imprecision of results.

### Knowledge

One RCT and one NRSI reported knowledge combined with attitudes scores [38, 42]. The results of Phillips 2017 for perceived knowledge and assessment tool suggested an effect in favour of the intervention measured directly after (MD -1.3, 95% CI: -2.1 to 0.6, *p <* 0.001 and MD -3.6, 95% CI: -0.5 to 2.2, *p <* 0.001; eTable 8) and at ten weeks after the intervention (MD -1.7, 95% CI: -2.2 to 1.1, *p <* 0.001 and MD -3.6, 95% CI: -0.5 to 2.2, *p <* 0.001; eTable 8), whereas little to no difference has been suggested between these time points (MD -0.3, 95% CI: -1.1 to 0.4 and MD 0.00, 95% CI: -0.7 to 0.7; eTable 8) [39]. Overall, the interventions may have little to no effect on knowledge, but the evidence is very uncertain evidence due to serious (for RCTs) and extremely serious (for NRSIs) limitations in the study design, serious indirectness and very serious imprecision.

### Certainty in the evidence

Table 3 provides the GRADE Evidence Profile including the detailed judgement of the certainty and the narrative summary of findings. Overall, the certainty in the evidence was judged to be low for overall survival, quality of life (evidence from RCTs), screening, referrals, prescribing behaviour (evidence from RCTs), and very low for all other outcomes (Table 3). The most frequent reasons for downgrading of the certainty were limitations in the study design, indirectness and imprecision of the results (Table 3).

**Table 3:** GRADE Evidence Profile Comparison: Multi-component guideline implementation intervention vs no intervention or usual care

Certainty assessment							Impact (narrative summary)	Certainty
**Study design**	**N**	**Risk of bias**	**Inconsistency**	**Indirectness**	**Imprecision**	**Other considerations**		
**Overall survival (patient-level outcome)**								
Randomized controlled trial	2	serious^a^	not serious^b^	not serious	serious^c^	none	The intervention may have little to no effect on overall survival	⨁⨁◯◯ Low
Non-randomized study	NA	NA	NA	NA	NA	NA	NA	NA
**Quality of life (patient-level outcome)**								
Randomized controlled trial	1	serious^a^	not serious^d^	not serious	serious^c^	none	The intervention may have little to no effect on pain reduction and overall quality of life scores	⨁⨁◯◯ Low
Non-randomized study	2	very serious^*a*^	not serious^*b*^	serious^*c*^	very serious^*d*^	none	The intervention may have little to no effect on pain intensity scores, but the evidence is very uncertain	⨁◯◯◯ Very low
**Adverse events (patient-level outcome)**								
Randomized controlled trial	2	serious^a^	serious^e^	not serious	serious^f^	none	The intervention may have little to no effect on adverse events, but the evidence is very uncertain	⨁◯◯◯ Very low
Non-randomized study	NA	NA	NA	NA	NA	NA	NA	NA
**Screening (provider-level outcome)**								
Randomized controlled trial	2	serious^a^	not serious	not serious	serious^f^	none	The intervention may increase screening rates according to guidelines	⨁⨁◯◯ Low
Non-randomized study	NA	NA	NA	NA	NA	NA	NA	NA
**Referral (provider-level outcome)**								
Randomized controlled trial	2	serious^a^	not serious^g^	not serious	serious^f^	none	The intervention may increase referral rates slightly according to guidelines	⨁⨁◯◯ Low
Non-randomized study	NA	NA	NA	NA	NA	NA	NA	NA
**Prescribing behaviour (provider-level outcome)**								
Randomized controlled trial	1	serious^a^	not serious^d^	not serious	serious^h^	none	The intervention may increase guideline adherence slightly regarding prescribing behaviour	⨁⨁◯◯ Low
Non-randomized study	2	very serious^*e*^	not serious^*f*^	not serious^*g*^	very serious^*h*^	none	The intervention may have little to no effect on prescribing behaviour, but the evidence is very uncertain	⨁◯◯◯ Very low
**Attitudes (provider-level outcome)**								
Randomized controlled trial	2	serious^a^	not serious^i^	serious^j^	very serious^k^	none	The intervention may have little to no effect on attitudes, but the evidence is very uncertain	⨁◯◯◯ Very low
Non-randomized study	4	very serious^*i*^	not serious^*f*^	serious^*l*^	very serious^*h*^	none	The intervention may have little to no effect on attitudes, but the evidence is very uncertain	⨁◯◯◯ Very low
**Knowledge (provider-level outcome)**								
Randomized controlled trial	1	serious^a^	not serious^d^	very serious^l^	very serious^m^	none	The intervention may have little to no effect on knowledge, but the evidence is very uncertain	⨁◯◯◯ Very low
Non-randomized study	2	extremely serious^*k*^	not serious^*f*^	serious^*l*^	very serious^*h*^	none	The intervention may have little to no effect on knowledge, but the evidence is very uncertain	⨁◯◯◯ Very low

GRADE Working Group grades of evidence

**High** = Further research is very unlikely to change the confidence in the estimate of effect

**Moderate** = Further research is likely to have an important impact on the confidence in the estimate of effect and may change the estimate

**Low** = Further research is very likely to have an important impact on the confidence in the estimate of effect and is likely to change the estimate

**Very low** = Any estimate of effect is very uncertain

*N* Number of studies, *NRSIs* Non-randomized studies of interventions (controlled before-and after studies), *NA* Not available, other considerations publication bias, *CIPN* Chemotherapy-induced peripheral neuropathy

**Explanations a-m (randomized controlled trials)**

^a^Downgraded one level for serious risk of bias, due to the lack of blinding of personnel, participants, and outcome assessors

^b^Results were not pooled but there could be heterogeneity between results explained by the different follow-ups and outcome definitions

^c^Downgraded one level for imprecision due to wide 95% confidence intervals and few participants

^d^Only one study reported this outcome

^e^Downgraded one level for inconsistency. Both studies suggested effects in completely opposite directions

^f^Downgraded one level for imprecision due to few events, participants and wide 95% confidence intervals

^g^Results were not pooled but there could be heterogeneity between results explained by the different outcome definitions, measurements and follow-ups

^h^Downgraded one level for imprecision due to few events

^i^One study did not report data adequately, therefore study results could not be compared. If there was heterogeneity between results, this may have arisen due to the different outcome definitions and measurements

^j^Downgraded one level for serious indirectness, because one study reported this outcome as a combined outcome: attitudes and knowledge, and not consistent with the definition of outcomes in this review (attitudes separately considered from knowledge). The other study measured attitudes and knowledge separately

^k^Downgraded two levels for very serious imprecision, due to few participants, few events, and wide 95% confidence intervals. One study did not adequately report the data, therefore an effect could not be further quantified

^l^Downgraded two levels for very serious indirectness, because the study reported this outcome as a combined outcome: attitudes and knowledge, and was not consistent with the definition of outcomes in this review (knowledge separately considered from attitudes)

^m^Downgraded two levels for very serious imprecision, because of few events and wide 95% confidence intervals

**Explanations *a-l* (non-randomized studies of interventions)**

^*a*^Downgraded for very serious risk of bias, due to the lack of control for confounders, deviations from intended intervention in one study, serious limitations in the outcome measurement due to the lack of blinding and poorly reported results (risk for reporting biases) found in these two studies

^*b*^The direction of the effect differs between studies due to heterogeneity in the population, outcome measurements, and follow-up times

^*c*^Downgraded one level for serious indirectness, because one study report only one symptom impacting quality of life (pain intensity)

^*d*^Downgraded two levels for very serious imprecision, because of few participants and wide 95% confidence intervals reported in both studies

^*e*^Downgraded two levels for very serious risk of bias, due to the lack of randomisation (confounders not controlled), inadequate selection of participants in one study, limitations in the outcome measurement, and due to the lack of blinding and poorly reported results (risk for reporting biases) found in these two studies

^*f*^The results show similar direction of effects (not pooled). Heterogeneity can be explained due to different outcome definitions and follow-ups

^*g*^The PICO framework of the studies addressed the review question

^*h*^Downgraded two levels for very serious imprecision, because of few participants, few events, and wide 95% confidence intervals in both studies

^*i*^Downgraded two levels due to the lack of randomisation and control for confounders in all NRSIs (one study with critical risk), limitations in the selection of participants of two studies, potential biases due to missing data, and biases in the measurement of outcomes (lack of blinding in all studies) and poorly reported outcomes in all studies

^*j*^Downgraded one level for serious indirectness, because one study measured attitudes and knowledge as a combined outcome. Another study measured attitudes only after intervention so that the comparison was not implemented as expected to see a difference before-and after the intervention

^*k*^Downgraded for extremely serious risk of bias, due to the overall critical risk of bias judged in one study and serious risk in the other study

^*l*^Downgraded one level for serious indirectness, because one study measured attitudes and knowledge as a combined outcome

## Discussion

A total of nine studies, five cluster RCTs and four controlled NRSIs with before-and after study design, were included in the synthesis [34–42]. All studies assessed multi-component guideline implementation interventions compared to no intervention in 3577 cancer patients and more than 450 oncologists, nurses and medical staff. Educational meetings combined with materials, opinion leaders, audit and feedback, a tailored intervention or academic detailing may have little to no effect on overall survival, quality of life and adverse events of cancer patients compared to no intervention, however, the evidence is either uncertain or very uncertain. Multi-component interventions may increase or slightly increase guideline adherence regarding screening, referral and prescribing behaviour of healthcare professionals according to guidelines, but the certainty in evidence is low. The interventions may have little to no effect on attitudes and knowledge of healthcare professionals, still, the evidence is very uncertain.

The present review confirms the findings from previous reviews in other clinical settings that educational strategies are the most frequently used strategies for guideline implementation and the most commonly used component in multi-component interventions [22–26, 57, 58]. Compared to the the review of Tomasone et al. [26], this review focuses on more recent literature. While Tomasone 2020 included 33 studies published between 1998 and 2018, we focused on more recent evidence up to December 2022. In contrast to the present review, Tomasone 2020 primarily focused on guideline adherence of healthcare professionals and secondarily on patient-relevant outcomes (survival, quality of life, test completion, pain) and concluded that the most used strategies were educational strategies and feedback on guideline compliance. In addition, the authors used a different taxonomy for coding the interventions, the Mazza taxonomy. This taxonomy builds upon the EPOC taxonomy but has implemented and adapted further domains [59].

A review in the dental care setting [57] showed that multi-component interventions showed slightly higher improvements in guideline adherence outcomes when compared to single interventions. In contrast to the present review, these reviews [57, 58] focused on other clinical settings and included studies conducted in other geographical locations. Moreover, these focused primarily on guideline adherence outcomes. Further, these reviews included a higher number of studies due to different inclusion criteria compared to the current review, such as wider time frames for publication of studies, the inclusion of further study designs (e.g. uncontrolled, retrospective), different classification of interventions, and different outcomes.

Some limitations of the included body of evidence in this review need to be mentioned. For instance, although authors of included studies that did not clearly describe the effect estimates were contacted, no response was obtained, affecting he completeness of the results. Only studies conducted in high-income countries, USA, Australia and France, were identified. Therefore, results may not apply to other countries, where different health systems, local values, and preferences exist. Also, the results may not be generalised to all cancer types or oncological settings. Due to the substantial clinical heterogeneity in participant characteristics, interventions, and outcomes, the pooling of results in meta-analyses was not feasible. This affected the ability of the synthesis to determine the quantitative effect of the interventions.

Potential weaknesses in the review process were that we did not search clinical trial registries due to time constraints, however, this mainly impacts the completeness of the list of ongoing studies and less the results of this review. The time restriction (studies published after 2011) may be interpreted as weakness, as otherwise fitting studies may have been excluded. However, the time frame was chosen to reflect more recent guideline implementation strategies. In addition, the categorisation of interventions conforming to the revised EPOC taxonomy [56] was done by one reviewer (AB) and did not follow a standardised algorithm. This subjective assessment could have introduced bias, as other reviewers could have differently classified the interventions into the predefined categories. The poor reporting of intervention details of some studies amplified the difficulty to classify strategies according to the taxonomy.

One of the strengths of the current review is its comprehensive electronic literature search in five databases, and additionall screening of references of relevant studies and relevant systematic reviews. The search in each database was optimised with the assistance of an experienced information specialist. Six ongoing trials were also included in the review to reflect the latest state of research in this area. Further, this review was conducted according to the recommendations of the Cochrane Handbook for Systematic Reviews of Interventions [28], of the PRISMA statement [29], in concordance with the EPOC taxonomy [56], and was previously registered in PROSPERO (CRD42021268593).

The effect of CPGs depends on how they are implemented and embedded in clinical practice [13, 16]. The implementation strategies suggested by the German Association of the Scientific Medical Societies (AWMF) coincide with the strategies identified by this review, namely interactive training, discussions, feedback, and local opinion leaders [16]. Moreover, in Germany, guideline-derived Quality Indicators (QIs) are used as key figures during cancer type-specific certification processes in order to determine whether a given center actually provides guideline-based treatment [60]. Here plays the interaction between guideline groups, QIs, certified centers and clinical cancer registries an important role, this being part of the German National Cancer Plan [61, 62]. Additional digital strategies of implementation found in this review, such as online education modules or digital monitoring of patient-reported outcomes, seem to be feasible in the current oncological setting [39, 41]. For instance, online-spaced learning involves the sending of short clinical case-based scenarios that take less than five minutes to consider, to participants’ e-mail or mobile device [39].

Although the evidence of this present review was rated to be low or very low, it does not necessarily mean that these multi-component strategies are not effective in oncology. According to the recommendations of the Institute of Medicine (IOM) and multiple behavioural change frameworks (e.g. COM-B-Behaviour Change Framework [63]), the effect may be amplified when combining many strategies that target the same or different patterns of behaviour change of health professionals and such interventions should be preferred [2]. The current preference for multi-component, professional-targeted interventions in oncology is in line with the implementation of CPGs in other settings [23, 57]. Also, the IOM recommends that “effective multi-component implementation strategies targeting both individuals and healthcare systems should be employed by implementers to promote adherence to trustworthy CPGs” [2]. However, the implementation of multi-component interventions can be demanding as two or more single strategies are involved. Especially, professional-targeted interventions may be hard to implement as behaviour patterns are always difficult to change, regardless of the setting. Moreover, strategies need to be in line with the high volume of recommendations and their frequent updates. This may require resources like time, money, and trained staff. As stated by the ESMO, without adoption in clinical routine practice, “even CPGs of the highest quality may be useless” [8]. In order to be successful, “CPGs have to be developed, disseminated to the right target audience, and finally be implemented” [8]. Facilitators of implementation of CPGs in oncology, found in a recent review, include the accessibility and ease of use of guidelines, dissemination of CPGs, adequate access to treatment facilities and resources, awareness of CPGs, belief in their relevance, and support in decision-making [64]. Furthermore, provider-related barriers such as behavioural patterns of health professionals, values, attitudes, and prior knowledge that affect the adaptability and adherence to changes should be addressed prior to the development of implementation strategies. The focus on local organisational structures, multidisciplinarity of the setting, availability of resources and support is essential when developing guideline implementation strategies. The development of complex and clearly reported interventions based on evidence-based theoretical frameworks may offer a greater potential for changing the clinical practice and a better understanding of barriers and facilitators of guideline implementation [65, 66].

**Fig. 4:**
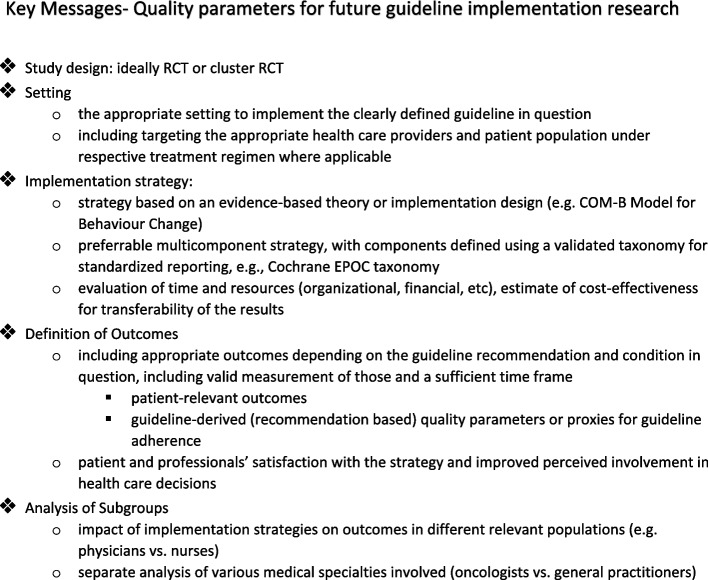
Key Messages Box for future guideline implementation research

We developed a list of quality parameters for future guideline implementation research that can be found in Fig. 4 (Key Messages Box). High-quality cluster randomized controlled trials and prospectively registered observational studies, designed to primarily assess patient-relevant outcomes emerging from changes in the behaviour of healthcare professionals, are needed. Future studies could consider the analysis of participants in subgroups, as the impact on the various clinical specialities involved in the oncological setting could differ (e.g. doctors vs nurses). In addition, a clear definition of the interventions is essential. The consistency in reporting of the implementation strategies according to a classification framework or taxonomy should be improved in future studies.

## Conclusions

The results should be interpreted with caution due to the low certainty of the evidence for overall survival, quality of life (in RCTs), screening, referrals, prescribing behaviour (in RCTs), and very low certainty of the evidence for all other outcomes. Team-oriented or online educational training and dissemination of materials embedded in multi-component interventions seem to be the most frequently researched strategies in the last years in oncology. This systematic review provides an overview of recent guideline implementation strategies in oncology, encourages future implementation research in this area and informs policymakers and professional organisations on the development and adoption of implementation strategies.

## Supplementary Information

**Additional file 1.** Search strategies. List of excluded studies at full-text screening stage. Population characteristics. Detailed description of interventions of included studies. Reported outcomes in the included studies. Characteristics of ongoing studies. Characteristics of studies awaiting classification. Risk of bias judgement for randomized controlled trials. Risk of bias judgement for non-randomized controlled studies of interventions. Risk of bias summary plots. Outcome effect tables. PRISMA Checklist.

## Data Availability

Available at request of the corresponding author.
