# Supplementary material for: The effectiveness of clinical guideline implementation strategies in oncology—a systematic review
Source: BMC Health Serv Res. 2023 Apr 6;23:347. doi: 10.1186/s12913-023-09189-x (PMC10080872; doi:10.1186/s12913-023-09189-x)
Supplement: Supplementary file 1 — Additional file 1. Search strategies. List of excluded studies at full-text screening stage. Population characteristics. Detailed description of interventions of included studies. Reported outcomes in the included studies. Characteristics of ongoing studies. Characteristics of studies awaiting classification. Risk of bias judgement for randomized controlled trials. Risk of bias judgement for non-randomized controlled studies of interventions. Risk of bias summary plots. Outcome effect tables. PRISMA Checklist. [file 12913_2023_9189_MOESM1_ESM.docx]

**Additional files**

[**Additional File 1. Search strategies** 1](#_Toc125620816)

[**Additional File 2. List of excluded studies at full-text screening stage** 5](#_Toc125620817)

[**Additional File 3. Population characteristics** 8](file:///V:\CHMG\Allgemein\2022_Implementation%20oncology\BMC%20Health%20Services%20Research\BMC_revised%20Supplementary%20Material_25_01_2023.docx#_Toc125620818)

[**Additional File 4. Detailed description of interventions of included studies** 10](#_Toc125620819)

[**Additional File 5. Reported outcomes in the included studies** 13](#_Toc125620820)

[**Additional File 6. Characteristics of ongoing studies** 15](#_Toc125620821)

[**Additional File 7. Characteristics of studies awaiting classification** 17](#_Toc125620823)

[**Additional File 8. Risk of bias judgement for randomized controlled trials** 20](#_Toc125620832)

[**Additional File 9. Risk of bias judgement for non-randomized controlled studies of interventions** 25](#_Toc125620833)

[**Additional File 10. Risk of bias summary plots** 31](#_Toc125620834)

[**Additional File 11. Outcome effect tables** 32](#_Toc125620835)

[**Additional File 12. PRISMA Checklist** 41](#_Toc125620836)

[**References** 44](#_Toc125620837)

This supplementary material has been provided by the authors to give readers additional information about their work

## **Additional File 1. Search strategies**

**PubMed** strategy (searched 16.12.2022)

| #1 Population: physicians, nurses, health workers) | "Health Personnel"[Mesh] OR "health worker*"[tiab] OR "physician*"[tiab] OR "Physicians"[Mesh] OR "nurse*"[tiab] OR "Nurses"[Mesh] OR "Oncologists"[Mesh] OR "oncologist*"[tiab] OR "health-care worker*"[tiab] OR "health-care practitioner*"[tiab] OR "health-care professional*"[tiab] OR "health-care staff"[tiab] OR "health-care personnel"[tiab] OR "health-care employee*"[tiab] OR "hospital worker*"[tiab] OR "hospital practitioner*"[tiab] OR "hospital professional*"[tiab] OR "hospital staff" [tiab] OR "hospital personnel" [tiab] OR "hospital employee*"[tiab] OR "medical worker*"[tiab] OR "medical staff"[tiab] OR "medical profession*"[tiab] OR "medical personnel" [tiab] OR "medical administrator*"[tiab] OR "doctor*"[tiab] OR "paramedic*"[tiab] OR "emergency medical technician*"[tiab] OR "emergency medical assistant*"[tiab] OR "medical student" [tiab] OR "anaesthesist*"[tiab] or anesthesiologist*[tiab] OR "cardiologist*"[tiab] OR "dentist*"[tiab] OR "dental practitioner*"[tiab] OR "dental staff" [tiab] OR "dental assistant*"[tiab] OR "general practitioner*"[tiab] OR "geriatrician*"[tiab] OR "internist*"[tiab] OR "neurosurgeon*"[tiab] OR "nephrologist*"[tiab] OR "neurologist*"[tiab] OR "ophthalmologist*"[tiab] OR "palliative care specialist*"[tiab] OR "palliative care staff" [tiab] OR "pharmacist*"[tiab] OR "psychiatrist*"[tiab] OR "pulmologist*"[tiab] OR "radiologist*"[tiab] OR "rheumatologist*"[tiab] OR "surgeon*"[tiab] OR "surgical staff"[tiab] |
| --- | --- |
| #2 Intervention: Guideline implementation strategies | "guideline*"[tiab] OR "clinical practice guideline*"[tiab] OR "Practice Guidelines as Topic"[Mesh] OR "Standard of Care"[Mesh] OR "Health Plan Implementation"[Mesh] OR "Health Plan Implementation*"[tiab] OR "Implementation Science"[Mesh] OR "Information Dissemination"[Mesh] OR "Information Dissemination" [tiab] OR "Information Distribution" [tiab] OR "Information Sharing" [tiab] OR "Data Sharing*"[tiab] OR "Translational Medical Science*"[tiab] OR "translational research, biomedical"[MeSH Terms] OR "Knowledge Translation*"[tiab] OR "Translational Research*"[tiab] OR "Translational Medicine"[tiab] OR "evidence-based"[tiab] OR "Evidence-Based Practice"[Mesh] OR "best practice"[tiab] |
| #3 Outcome: survival, quality of life, safety, guideline adherence, screening rate, referral, prescribing behaviour, attitude, knowledge | "survival"[tiab] OR "Survival"[Mesh] OR "quality of life"[tiab] OR "Quality of Life"[Mesh] OR "personal satisfaction" [tiab] OR "patient satisfaction" [tiab] OR "Personal autonomy"[tiab] OR "Happiness" [tiab] OR "positive experience"[tiab] OR "safety"[tiab] OR "Safety"[Mesh] OR "harm"[tiab] OR "Patient Harm"[Mesh] OR "guideline adherence" [tiab] OR "Guideline Adherence"[Mesh] OR "compliance" [tiab] OR "referral*" [tiab] OR "Referral and Consultation"[Mesh] OR "screening*" [tiab] OR "Early Detection of Cancer"[Mesh] OR "Diagnosis"[Mesh] OR "Drug Prescriptions"[Mesh] OR "Attitude"[Mesh] OR "Health Knowledge, Attitudes, Practice"[Mesh] OR "Attitude of Health Personnel"[Mesh] OR "Knowledge"[Mesh] OR "behaviour" [tiab] |
| #4 Setting: cancer care | "cancer" [tiab] OR "malignant tumour" [tiab] OR "Neoplasms"[Mesh] OR "neoplas*"[tiab] OR "tumor*"[tiab] OR "tumour*"[tiab] OR "malignan*"[tiab] OR "carcino*" [tiab] OR "karzinom*"[tiab] OR "sarcom*"[tiab] OR "leukem*"[tiab] OR "leukaem*" [tiab] OR "lymphom*"[tiab] OR "melano*"[tiab] OR "metastas*"[tiab] OR "gliom*"[tiab] OR "glioblastom*"[tiab] OR "osteosarcom*"[tiab] OR "blastom*"[tiab] OR "neuroblastom*"[tiab] OR "myeloma*"[tiab] OR "Medical Oncology"[Mesh] OR "oncology" [tiab] |
|  | #1 AND #2 AND #3 AND #4 |
| Additional Filters | Filters: Randomized Controlled Trial, from 2011 - 2022 |

**Web of science** strategy (searched 16.12.2022)

| #1 Intervention: Guideline implementation strategies | TS=((guideline* OR clinical practice guideline* OR recommendation* OR standard* OR evidence-based practice OR clinical practice)AND (implementation* OR dissemination* OR guideline implementation OR implementation science)) |
| --- | --- |
| #2 Population:  physicians, nurses, health workers) | TS=(health personnel OR health worker* OR healthcare professional* OR physician* OR nurse* OR oncologist*) |
| #3 Setting:  cancer care | TS=(cancer* OR neoplasm* OR tumour* OR malignan* OR oncolog* OR cancer guideline* OR medical oncology) |
| #4 Outcome:  survival, quality of life, safety, guideline adherence, screening rate, referral, prescribing behaviour, attitude, knowledge | TS=(survival* AND quality of life AND safety OR adverse events OR harm* AND guideline adherence OR compliance AND behaviour OR physicians behaviour AND attitudes AND referrals AND screening rate* AND prescribing behaviour) |
| #5 Additional filter | **2021** or **2020** or **2019** or **2018** or **2017** or **2016** or **2015** or **2014** or **2013** or **2012** or **2011** (Publication Years) |
|  | #1 AND #2 AND #3 AND #4 AND #5 |

**CENTRAL** simple search (searched 16.12.2022): *guideline implementation strategies in oncology* (Tittle Abstract Keyword) + additional time filter

**GIN** database search pathway (searched 16.12.2022):

GIN-> GIN Conference -> Previous years-> Abstracts

- 2016 GIN Philadelphia

Search every presentation of each conference for potential eligible studies

- 2018 GIN Manchester
- 2019 Adelaide
- 2021 Online
- 2022 Toronto

**CINAHL (via EBSCO)** (searched 16.12.2022)

| **#** | **Query** |
| --- | --- |
| S60 | S59 Limiters - Published Date: 20110101-20210931 |
| S59 | S43 AND S58 |
| S58 | S51 NOT S57 |
| S57 | S55 NOT S56 |
| S56 | MH human |
| S55 | S52 OR S53 OR S54 |
| S54 | TI animal model* |
| S53 | MH animal studies |
| S52 | MH animals+ |
| S51 | S44 OR S45 OR S46 OR S47 OR S48 OR S49 OR S50 |
| S50 | AB cluster W3 RCT |
| S49 | MH crossover design OR MH comparative studies |
| S48 | AB control W5 group |
| S47 | PT randomized controlled trial |
| S46 | MH placebos |
| S45 | MH sample size AND AB ( assigned OR allocated OR contro ) |
| S44 | MH randomized controlled trial OR MH double-blind studies OR MH single-blind studies OR MH random assignment OR MH pretest-posttest design OR MH cluster sample OR TI ( randomised OR randomized ) OR TI random* OR TI trial |
| S43 | S10 AND S20 AND S26 AND S37 AND S42 |
| S42 | S38 OR S39 OR S40 OR S41 |
| S41 | TX melano* OR TX metastas* OR TX gliom* OR TX glioblastom* OR TX osteosarcom* OR TX blastom* OR TX neuroblastom* OR TX myeloma* OR TX oncology |
| S40 | TX cancer OR TX malignan* OR TX neoplas* OR TX tumor* OR TX tumour* OR TX carcino* OR TX karzinom* OR TX sarcom* OR TX leukem* OR TX leukaem* OR TX lymphom* |
| S39 | (MH "Neoplasms+") |
| S38 | (MH "Oncology+") |
| S37 | S27 OR S28 OR S29 OR S30 OR S31 OR S33 OR S34 OR S35 OR S36 |
| S36 | TX guideline adherence OR TX compliance OR TX referral* OR TX screening* OR TX behaviour OR TX behavior |
| S35 | (MH "Behavior+") |
| S34 | (MH "Health Knowledge") |
| S33 | (MH "Attitude of Health Personnel+") |
| S32 | (MH "Attitude+") |
| S31 | (MH "Prescriptions, Drug+") |
| S30 | (MH "Diagnosis+") |
| S29 | (MH "Early Detection of Cancer") |
| S28 | (MH "Referral and Consultation+") |
| S27 | (MH "Guideline Adherence") |
| S26 | S21 OR S22 OR S23 OR S24 OR S25 |
| S25 | TX survival OR TX quality of life OR TX personal satisfaction OR TX patient satisfaction OR TX Personal autonomy OR TX Happiness OR TX positive experience OR TX safety OR TX harm |
| S24 | (MH "Quality of Life+") |
| S23 | (MH "Harm Reduction") |
| S22 | (MH "Safety+") |
| S21 | (MH "Survival") |
| S20 | S13 OR S14 OR S15 OR S16 OR S17 OR S18 OR S19 |
| S19 | TX guideline* OR TX Health Plan Implementation* OR TX Information Dissemination OR TX Information Distribution OR TX Information Sharing OR TX Data Sharing* OR TX Translational Medical Science* OR TX Knowledge Translation* OR TX Translational Research* OR TX Translational Medicine OR TX evidence-based OR best practice |
| S18 | (MH "Selective Dissemination of Information") |
| S17 | (MH "Translational Medical Research") |
| S16 | (MH "Implementation Science") |
| S15 | (MH "Practice Guidelines") |
| S14 | (MH "Professional Practice, Evidence-Based+") |
| S13 | S11 AND S12 |
| S12 | (MH "Program Implementation") |
| S11 | (MH "Health and Welfare Planning+") |
| S10 | S1 OR S2 OR S3 OR S4 OR S5 OR S6 OR S7 OR S8 OR S9 |
| S9 | TX surgical staff |
| S8 | TX neurosurgeon* OR TX nephrologist* OR TX neurologist* OR TX ophthalmologist* OR TX palliative care specialist* OR TX palliative care staff OR TX pharmacist* OR TX psychiatrist* OR TX pulmologist* OR TX radiologist* OR TX rheumatologist* OR TX surgeon* |
| S7 | TX emergency medical assistant* OR TX medical student OR TX anaesthesist* OR TX anesthesiologist* OR TX cardiologist* OR TX dentist* OR TX dental practitioner* OR TX dental staff OR TX dental assistant* OR TX general practitioner* OR TX geriatrician* OR TX internist* |
| S6 | TX hospital professional* OR TX hospital staff OR TX hospital personnel OR TX hospital employee* OR TX medical worker* OR TX medical staff OR TX medical profession* OR TX medical personnel OR TX medical administrator* OR TX doctor* OR TX paramedic* OR TX emergency medical technician* |
| S5 | TX health worker* OR TX physician* OR TX nurse* OR TX oncologist* OR TX health-care worker* OR TX health-care practitioner* OR TX health-care professional* OR TX health-care staff OR TX health-care personnel OR TX health-care employee* OR TX hospital worker* OR TX hospital practitioner* |
| S4 | TX health worker* |
| S3 | (MH "Nurses+") |
| S2 | (MH "Physicians+") |
| S1 | (MH "Health Personnel+") |

## **Additional File 2. List of excluded studies at full-text screening stage**

| **Excluded references** | **Primary reason for exclusion** |
| --- | --- |
| A. E. Maxwell, L. L. Danao, R. T. Cayetano, C. M. Crespi, and R. Bastani, "Implementation of an evidence-based intervention to promote colorectal cancer screening in community organizations: a cluster randomized trial," (in eng), Transl Behav Med, vol. 6, no. 2, pp. 295-305, Jun 2016, doi: 10.1007/s13142-015-0349-5. | **I** |
| A. Morrow et al., Building capacity from within: qualitative evaluation of a training program aimed at upskilling healthcare workers in delivering an evidence-based implementation approach. Transl Behav Med. 2022 Jan 18;12(1):ibab094. doi: 10.1093/tbm/ibab094. | **I** |
| C. M. Witt et al., "Training oncology physicians to advise their patients on complementary and integrative medicine: An implementation study for a manual-guided consultation," (in eng), Cancer, vol. 126, no. 13, pp. 3031-3041, Jul 1 2020, doi: 10.1002/cncr.32823. | **D** |
| C. Rat et al., "Effect of Physician Notification Regarding Nonadherence to Colorectal Cancer Screening on Patient Participation in Fecal Immunochemical Test Cancer Screening: A Randomized Clinical Trial," (in eng), Jama, vol. 318, no. 9, pp. 816-824, Sep 5 2017, doi: 10.1001/jama.2017.11387. | **I** |
| D. Hountz, J. Coddington, K. J. Foli, and J. Thorlton, "Increasing Colorectal Cancer Screening Using a Quality Improvement Approach in a Nurse-Managed Primary Care Clinic," (in eng), J Healthc Qual, vol. 39, no. 6, pp. 379-390, Nov/Dec 2017, doi: 10.1097/jhq.0000000000000107. | **D** |
| G. Samuelly-Leichtag, T. Adler, and E. Eisenberg, "Something Must Be Wrong with the Implementation of Cancer-pain Treatment Guidelines. A Lesson from Referrals to a Pain Clinic," (in eng), Rambam Maimonides Med J, vol. 10, no. 3, Jul 18 2019, doi: 10.5041/rmmj.10369. | **I** |
| I. Gessl et al., "Surveillance colonoscopy in Austria: Are we following the guidelines?," (in eng), Endoscopy, vol. 50, no. 2, pp. 119-127, Feb 2018, doi: 10.1055/s-0043-119637. | **D** |
| I. Litchfield, S. Greenfield, GM. Turner, S. Finnikin, MJ. Calvert. Implementing PROMs in routine clinical care: a qualitative exploration of GP perspectives. BJGP Open. 2021 Feb 23;5(1):bjgpopen20X101135. doi: 10.3399/bjgpopen20X101135. | **D** |
| J. Alfieri et al., "Development and impact evaluation of an e-learning radiation oncology module," (in eng), Int J Radiat Oncol Biol Phys, vol. 82, no. 3, pp. e573-80, Mar 1 2012, doi: 10.1016/j.ijrobp.2011.07.002. | **I** |
| M. Weslau et al., OncoCoaching and early palliative care for interprofessional and patient-centered care in the therapy of incurable cancer-Project OnCoPaTh. Jahrestagung der Deutschen, Österreichischen und Schweizerischen Gesellschaften für Hämatologie und Medizinische Onkologie, 01. - 04. Oktober 2021, Berlin. doi:10.1159/000518417. | **I** |
| MB. Marzal-Alfaro et al., Error Detection and Cost Savings With an Image-Based Workflow Management System Connected to a Computerized Prescription Order Entry Program for Antineoplastic Compounding. J Patient Saf. 2021 Dec 1;17(8):e1589-e1594. doi: 10.1097/PTS.0000000000000591. | **I** |
| N. Koesel, C. Tocchi, L. Burke, T. Yap, and A. Harrison, "Symptom Distress: Implementation of Palliative Care Guidelines to Improve Pain, Fatigue, and Anxiety in Patients With Advanced Cancer," (in eng), Clin J Oncol Nurs, vol. 23, no. 2, pp. 149-155, Apr 1 2019, doi: 10.1188/19.Cjon.149-155. | **I** |
| R. Knoerl, C. Bridges, G. L. Smith, J. J. Yang, G. Kanzawa-Lee, and E. M. L. Smith, "Chemotherapy-Induced Peripheral Neuropathy: Use of an Electronic Care Planning System to Improve Adherence to Recommended Assessment and Management Practices," (in eng), Clin J Oncol Nurs, vol. 22, no. 5, pp. E134-e140, Oct 1 2018, doi: 10.1188/18.Cjon.E134-e140. | **D** |
| RJ. Chan et al., Partnering with general practitioners to optimize survivorship for patients with lymphoma: a phase II randomized controlled trial (the GOSPEL I trial). Trials. 2021 Jan 6;22(1):12. doi: 10.1186/s13063-020-04945-4. | **I** |
| S. Lindsey, K. Astroth, and P. Kumar, "Improving Awareness, Identification, and Management of Sarcopenic Obesity in Cancer Survivors: An Evidence-Based Toolbox," (in eng), Clin J Oncol Nurs, vol. 20, no. 5, pp. E132-8, Oct 1 2016, doi: 10.1188/16.Cjon.E132-e138. | **I** |
| S. Sendelbach, K. E. Sandau, L. Smith, R. Kreiger, S. Hanovich, and M. Funk, "Implementing Practice Standards for Inpatient Electrocardiographic Monitoring," (in eng), Am J Crit Care, vol. 28, no. 2, pp. 109-116, Mar 2019, doi: 10.4037/ajcc2019699. | **D** |
| S. X. Raj, C. Brunelli, P. Klepstad, and S. Kaasa, "COMBAT study - Computer based assessment and treatment - A clinical trial evaluating impact of a computerized clinical decision support tool on pain in cancer patients," (in eng), Scand J Pain, vol. 17, pp. 99-106, Oct 2017, doi: 10.1016/j.sjpain.2017.07.016. | **I** |
| T. J. Huang, P. F. Mu, M. B. Chen, and K. Florczak, "Prevention and treatment of oral mucositis among cancer patients in the hematology-oncology setting: a best practice implementation project," (in eng), Int J Evid Based Healthc, Jul 17 2020, doi: 10.1097/xeb.0000000000000238. | **D** |
| Tian L, Yang Y, Sui W, Hu Y, Li H, Wang F, Qian K, Ji J, Tao M. Implementation of evidence into practice for cancer-related fatigue management of hospitalized adult patients using the PARIHS framework. PLoS One. 2017 Oct 31;12(10):e0187257. doi: 10.1371/journal.pone.0187257. PMID: 29088266; PMCID: PMC5663504 | **D** |
| W. Q. Chong, M. J. Mogro, A. Arsad, B. C. Tai, and S. C. Lee, "Use of decision aid to improve informed decision-making and communication with physicians on the use of oral complementary and alternative medicine (CAM) among cancer patients on chemotherapy treatment: a randomised controlled trial," (in eng), Support Care Cancer, vol. 29, no. 7, pp. 3689-3696, Jul 2021, doi: 10.1007/s00520-020-05872-5. | **I** |
| X. Su et al., "Effects of Evidence-Based Continuing Care Bundle on Health Outcomes in Rectal Cancer Patients With Temporary Stomas: A Multicenter Randomized Controlled Trial," (in eng), Cancer Nurs, vol. 44, no. 3, pp. 223-234, May-Jun 01 2021, doi: 10.1097/ncc.0000000000000762. | **I** |
| Y. Liu, L. Mo, Y. Tang, Q. Wang, and X. Huang, "The Application of an Evidence-Based Clinical Nursing Path for Improving the Preoperative and Postoperative Quality of Care of Pediatric Retroperitoneal Neuroblastoma Patients: A Randomized Controlled Trial at a Tertiary Medical Institution," (in eng), Cancer Nurs, vol. 40, no. 4, pp. 314-322, Jul/Aug 2017, doi: 10.1097/ncc.0000000000000387. | **I** |
| Y. Schenker et al., Effect of an Oncology Nurse-Led Primary Palliative Care Intervention on Patients With Advanced Cancer: The CONNECT Cluster Randomized Clinical Trial. JAMA Intern Med. 2021 Nov 1;181(11):1451-1460. doi: 10.1001/jamainternmed.2021.5185. | **I** |

D= ineligible study design (e.g. uncontrolled or retrospective design); I= ineligible intervention (e.g. no guideline implementation strategy).

| Study **Additional File 3. Population characteristics** | Participating providers | | | | | | | Patients | | | | | Clinical problem |
| --- | --- | --- | --- | --- | --- | --- | --- | --- | --- | --- | --- | --- | --- |
|  | **Clinical speciality** | | | **Work experience (years)** | **Age**  **(years)** | **Gender**  **(%)** | **N** | **Cancer type*** | **Age (years)** | **Sex**  **(% male)** | **N** | |  |
| Randomized controlled trials | | | | | | | | | | | | | |
| Brown  2018 | Urologists | | | NR | NR | NR | 37 | Prostate | Median 65 | 100% | 1071 | | Appropriate utilisation of adjuvant radiotherapy for men with locally advanced prostate cancer |
| Gilbert 2021 | Surgical ward health professionals | | | NR | NR | NR | NR | Colorectal cancer (CRC) | Median 79.6 | 50.3% | 147 | | Appropriate perioperative geriatric nutritional management according to guidelines |
| Lovell 2022 | Oncological and palliative care medical staff | | | NR | NR | NR | NR | Advanced cancers (Breast, Lung, Gastro, Genitourinary, Head and Neck, others) | Mean (SD):  Control: 64.2 (12.1)  Intervention: 63.6 (12.7) | 55% control  47% intervention | 688 | | Cancer Pain |
| McCarter 2018 | Oncology dieticians | | | NR | NR | NR | 29 | Head and neck (oropharynx) | Median 58 | 76% - 83% | 307 | | Malnutrition in adult head and neck cancer (HNC) patients undergoing radiotherapy |
| Mohile 2021 | Oncologists | | | NR | NR | NR | 156 | Incurable advanced solid tumours (Breast, Lung, Gastro, other) or lymphoma | Mean (SD):  77.2 (5.4) | 47% | 718 | | Reducing the risk for treatment toxic effects in geriatric advanced cancer patients |
| Non-randomized controlled studies of intervention | | | | | | | | | | | | | |
| Bonkowski 2018 | | Surgical oncology nurses | less than 1 year: 36%  1-5 years: 56%  5-10 years: 8% | | Mean 36.76 | 96% female | 26 | NR | NR | NR | 44 | Nurses lack adequate pain management knowledge, which can result in poorly managed postsurgical pain | |
| Cowperth-waite 2019 | | Oncology nurses | Mean (SD)  Pre: 16.7 (11.8)  Post: 9.4 years (12.3)  Unit experience:  Pre: 13.8 years (9)  Post: 7.9 years (9.8) | | Mean (SD) Pre: 42.5 (SD 12.1)  Post: 37.2 (SD 13.6) | NR | Pre: 11  Post: 9 | Pancreas, liver or gall-bladder, sarcoma, colon or gastric | Mean (SD)  Pre: 58.7 (14.96)  Post: 57.6 (13.03) | Pre: 52%  Post: 55% | Pre: 173  Post: 157 | Inadequate management of cancer pain denies comfort and acceptable quality of life, and may even reduce survival. | |
| Knoerl  2021 | | 54.7% physicians, 39.6% nurses, 5.7% physician assistants | breast (45.3%), gastrointestinal  (34%), and multiple myeloma (20.7%) outpatient centers | | Mean  44 | female: 62.3% | 53 | Breast, gastro-intestinal | Mean  57 | 33.8% | 142 | Chemotherapy-induced peripheral neuropathy (CIPN) is a frequent dose-limiting complication of neurotoxic  chemotherapy and it is under assessed and managed by clinicians | |
| Phillips 2017 | | Oncology Nurses  (61% ) | 43% with 6-15 years | | Mean  33.5 | 87% female | 125 | Lung, breast, gynaecological | Range  56-66 | 40% - 53% | 105-130 | Cancer Pain | |

NR= not reported; *= the most frequent type(s).

## **Additional File 4. Detailed description of interventions of included studies**

***Randomized controlled trials (RCTs)***

Brown 2018 (1) implemented the *Clinician-Led Improvement in Cancer Care (CLICC)* intervention, which was informed by a conceptual program logic model based on the PRECEDE-PROCEED theoretical framework of behaviour change (2). This theory focuses on the identification of barriers and „predisposing factors” (e.g., knowledge and attitudes of the target group) for these barriers. Therefore, this was categorised as *tailored intervention* according to EPOC. Afterwards, “reinforcing factors” (e.g., opinions and behaviour of peers) are identified. Moreover, the CLICC intervention included the following physician-focused components categorised according to the EPOC taxonomy (3):

- *Local opinion leaders*: Peer-identification of local Clinical Leaders, linked to the Urology Network and national opinion leaders, to reinforce key messages, model targeted referral behaviours and promote practice change (reinforcing factor).
- *Educational meeting*: Peer-to-peer education, facilitated by the local Clinical Leader, was implemented, including a video summary of the evidence underlying the clinical practice recommendation and the introduction of key messages through discussion of best clinical practice by state and national opinion leaders, and patient experiences of care (predisposing factor).
- Dissemination of *educational materials*, including the full clinical practice guideline, a quick reference guide, and supporting randomized controlled trial publications (predisposing factor).
- *Audit and feedback*: Quarterly *audit and feedback* report of individual clinicians’ practice (written feedback) and study sites’ aggregated practice (written and verbal feedback by the Clinical Leader) (reinforcing factor).

Gilbert 2021 (4) implemented a geriatric intervention which included an outreach geriatric team visiting surgical centers to provide training and advice to the health professionals focused on nutritional screening and management of undernutrition in accordance with prevailing guidelines. This implementation intervention included: scheduled meetings with the staff (*educational meetings*), regular visits to the wards (*academic detailing*), and the dissemination of written, informative documents (*educational materials*) to both patients and professionals.

Lovell 2022 (5) implemented an intervention developed using the Behaviour Change Wheel conceptual framework (6) and included the following components categorised according to the EPOC taxonomy:

- Opinion leader: Staff was given an overview of the guidelines and implementation resources by a local clinical champion with support from the project team.
- *Audit and feedback*: audit of adherence to 6 key guideline recommendations and feedback delivered in 1 to 2 cycles.
- *Educational meetings*: using email-administered spaced education via the Qstream platform.
- *Educational materials*: education booklet and a patient self-management resource.

McCarter 2018 (7) implemented the *EAT (Eating as treatment)* intervention, which is a behaviour change counselling intervention, which was aligned with six clinical practice guideline recommendations to ensure sufficient exposure of patients to the dietician-delivered intervention. This intervention was not informed by a theoretical framework of implementation. The intervention consisted of the following components categorised according to the EPOC taxonomy (3):

- *Educational meeting:* Staff training delivered in a two-day workshop.
- *Academic detailing* consisting of trainers that accompany dieticians during their usual consultations to assist the clinical implementation of EAT. The trainers returned within two months to refresh EAT intervention skills.
- *Educational materials:* nutrition assessment and depression-screening tools, which were implemented based on the lack of information and clinical uncertainty identified at the beginning of the intervention.
- *Audit and feedback*: Performance *audit and feedback* was implemented providing on- site performance based on agreed benchmarks in written reports and during telephone contacts every three to four months.

Mohile 2021 (8) used a geriatric assessment intervention which included cancer treatment considerations (eg, dose reduction in cycle one with escalation as tolerated), through literature review, guidelines, and expert consensus. Staff generated a tailored geriatric assessment summary and management recommendations using a web-based platform (*educational materials*). At study entry, oncologists in the intervention practices received brief training about geriatric assessment (*educational meetings*). Training provided an overview of how the geriatric assessment summary could be used to guide treatment decisions and how recommendations could be used to guide management of ageing-related conditions.

***Non-randomized controlled studies of interventions (NRSIs)***

Bonkowski 2018 (9) based their inpatient intervention in surgical oncology on the Knowledge-to-Action (KTA) framework (10). This model involves two parts: knowledge creation (knowledge inquiries, synthesizing information, and creating tools) and the action cycle (problem identification, selection of knowledge to use, adaptation to the situation, assessment of barriers to knowledge use, selection and implementation of interventions, monitoring and outcome evaluation). Knowledge creation was based on finding the latest evidence-based recommendations for postoperative pain management. The first component of the intervention was a *tailored intervention* because the implementation strategy was based on tailoring and seeking input from nurses on barriers and facilitators of their pain management (3). Two other strategies were combined with this tailored intervention, namely *educational meetings and the* dissemination of *educational materials.* These included a 20-minute online pain education module, a 30-minute live education session, and the dissemination of printed information regarding clinical practice recommendations on pain management.

Cowperthwaite 2019 (11) evaluated the implementation of a bundled evidence-based Pain-Stoppers intervention informed by the Ottawa Model of Research Use translation framework (12), which uses a six-step approach to guide the implementation of innovation: 1. Set the stage (determine resources for implementation); 2. Specify the innovation; 3. Assess the innovation (identify barriers, perceptions); 4. Select and monitor the knowledge translation strategies (appropriate strategies and intervention to increase awareness of the innovation); 5. Monitor innovation adoption; and 6. Evaluate outcomes of the innovation. Due to the lack of information on the prior collection of barriers in this intervention, it could not be categorised as a tailored intervention, as the other theory-based interventions. The Pain-Stoppers included communication, caring behaviours, timely responses, patient education and maintenance of analgesic levels (11). This was categorised as *patient-mediated intervention* (3), as professionals' practice change was targeted by closely interacting with patients. Additionally, nurses were provided with printed algorithm handouts delivered during discussion sessions, categorised as *educational meetings* and *educational materials* (3).

Knoerl 2021 (13) included the implementation of an evidence-based decision support algorithm for the management and assessment of the chemotherapy-induced peripheral neuropathy (CIPN), which incorporates evidence-based CIPN assessment of patients by using standardized patient-reported outcomes and management. This intervention used no theoretical framework of implementation. The implementation intervention consisted of two components (3): *educational materials* (i.e., algorithm handouts, CIPN clinical practice guideline, deep-tendon reflexes and vibration sensibility training video, patient-friendly resources about neuropathy safety), and *educational meeting* in form of a training session conducted by the principal investigator on how to use the algorithm.

The intervention implemented by Phillips 2017 (14) was based on the COM-B (Capability, Opportunity, Motivation— Behavioural) system framework to better understand and target the desired behaviour change in oncology nurses (14, 15). The intervention combined a so-called online-spaced cancer pain assessment learning module with a targeted behavioural change strategy, an audit and feedback of cancer pain assessment practices at each site, categorised as *educational meetings*, *educational materials* and *audit and feedback* (3). Eleven case-based cancer pain assessment scenarios were delivered directly to participants’ e-mail in a spaced, repeated, and tested format over 28 days via the automated QStream Internet platform. Spaced learning differs significantly from other learning methods because it sends short clinical case-based scenarios that take less than five minutes to consider to participants’ e-mail or hand-held mobile devices. Upon answering a question, participants’ performance was compared to their peers’ responses, and they were provided with succinct feedback and links to relevant evidence-based resources and decision supports.

## **Additional File 5. Reported outcomes in the included studies**

| **Study** | **Objective outcome(s)** | **Subjective outcome(s)** |
| --- | --- | --- |
| **Brown 2018** | Referral  (*referral within 4 months after RP (Radical prostatectomy*) | Knowledge and Attitudes* |
| **Gilbert 2021** | Screening  (*appropriate nutritional assessment*)  Adverse events (*patients with at least one AE, patients with at least one postsurgical outcome*)  Overall survival (30 days post-surgery) | - |
| **Lovell 2022** | - | Quality of life  (*patients with* *a score of 5 or more out of 10 on a worst pain numeric rating scale (NRS) with a pain reduction of 30% from the initial NRS at week 1, worst NRS pain score, total QLQ-C15-PAL score*) |
| **McCarter 2018** | Screening  (*depression screening at 1 week of RT (radiotherapy*),  Referral  (*depression referral at Week 1 of RT (radiotherapy*) | Attitudes  (*helpfulness of the practice change strategies*) |
| **Mohile 2021** | Adverse events  (*prevalence of any grade 3–5 Common Terminology Criteria for Adverse Events toxic effects over 3 months*)  Prescribing behaviour  (*number of overall medications discontinued before starting cancer treatment regimen, reduced dose intensity at cycle 1, dose modification at 3 months, relative dose intensity*)  Overall Survival  (*survival over 1 year*) | - |
| **Bonkowski 2018** | Prescribing behaviour  (*narcotic administrations 24 hours before discharge at 3 months after intervention by reviewing patient charts*) | Prescribing behaviour  (*changes in nursing pain management practice at six weeks after intervention using survey- items with 5-point Lickert Scale rating (only item 17 and 18 relevant*),  Attitudes |
| **Cowperthwaite 2019** | - | Quality of life  (*pain intensity measured with a scale ranging from 0 (no pain) to 10 (worst pain imaginable*) |
| **Knoerl 2021** | Prescribing behaviour  (*frequency of overall appropriate, appropriate mild and moderate-severe CIPN management using chart reviews*) | Quality of life  (*sensory and motor CIPN severity and worst CIPN pain intensity using the EORTC QLQ-CIPN20 Scale and the Worst CIPN Numerical Rating Scale*),  Knowledge and Attitudes*  (*mean number of correctly answered questions in the KAP (Knowledge, Attitudes, and Practices) survey and Acceptability and Feasibility scores 1 year after the intervention*) |
| **Phillips 2017** | Knowledge  (*pain assessment chart audit changes directly and one month after audit and feedback phase*) | Knowledge  (*perceived knowledge and assessment tool using a 11-point visual analogue rating scale directly after and 10 weeks after the intervention*)  Attitudes  (*confidence measured with an 11-point visual analogue rating scale at two time points (directly after the intervention and 10 weeks after the intervention*) |

Objective= objectively/ not self-reported/ outcome assessed by another person; Subjective= self-reported/ subjectively assessed outcome; *Knowledge and Attitudes- as one, combined outcome

## **Additional File 6. Characteristics of ongoing studies**

| Study  ID | Title | Links | Design | Status | Nr. of participants/ patients/ clusters | Intervention & comparator | Outcomes | Results available | Country,  Setting |
| --- | --- | --- | --- | --- | --- | --- | --- | --- | --- |
| NTR4058 | Stepped implementation of Enhanced Recovery After Surgery in major gynaecological surgery | <https://www.trialregister.nl/trial/3896>  <https://pubmed.ncbi.nlm.nih.gov/26223232/> | stepped-wedge  cluster RCT | Planned  Expected completion date: 11.01.2014 | 14 clusters | *Multi-component*: educational meetings, educational outreach visits, audit and feedback, opinion leaders  vs. active control (educational meetings) | Postoperative hospital stay, use of prophylactic antiemetics, use of epidural analgesia, use of laxatives | No | Netherlands,  inpatient |
| ACTRN12615000064505 | The Stop Cancer PAIN Trial: A guideline implementation study | <https://www.anzctr.org.au/Trial/Registration/TrialReview.aspx?id=367236&isReview=true>  <https://pubmed.ncbi.nlm.nih.gov/30012122/> | stepped-wedge  cluster RCT | Recruiting  Expected completion date: NR | 800 patients (≥ 60% with breast cancer) | *Multi-component*: tailored intervention, educational meetings and materials, audit and feedback  vs. active control (pain screening system) | QoL, pain screening improvement, patient empowerment | No | Australia,  outpatient |
| NCT04389502 | Efficacy of a Mobile Clinical Decision Support System (CaPtyVa CCR App) to Improve Performance of Gastroenterology and Coloproctology Specialists in Colorectal Cancer Screening and Surveillance According to Guideline Recommendations: A Randomized Clinical Trial. | <https://clinicaltrials.gov/ct2/show/NCT04389502?term=NCT04389502&draw=2&rank=1> | RCT | Recruiting  Expected completion date:  01.06.2020 | 200 participants | *Single-component*:  CaPtyVa CCR digital app-Mobile Decision Support System  vs. active control (10-item clinical vignette quiz) | Knowledge tested in clinical vignettes | No | Argentina,  inpatient |
| NCT04208490 | Implementation and Effectiveness Trial of HN-STAR (HN-STAR) | <https://clinicaltrials.gov/ct2/show/NCT04208490?term=NCT04208490&draw=2&rank=1>  <https://doi.org/10.1016/j.cct.2021.106448> | RCT | Recruiting  Expected completion date: 28.02.2024 | 470 patients, ≥ 30 oncology practices | *Single-component*:  Web-based tool „The Head and Neck Survivorship Tool (HN-STAR)”  vs. no intervention | QoL, Change in symptom burden,  Adherence and surveillance of guideline concordant care | No | USA,  inpatient |
| NTR2739 | Implementing the Dutch guideline "Pain in Cancer Patients" | <https://www.trialregister.nl/trial/2611>  <http://www.implementationscience.com/content/6/1/126> | cluster RCT | Planned  Expected completion date: 15.12.2014 | 210 participants within 6 clusters | *Multi-component*: SMS-IVR intervention, educational meetings and materials, patient-mediated intervention  vs. no intervention | QoL, pain intensity, neuropathic pain, percentage of patients with adequate pain medication | No | Netherlands,  outpatient |
| ACTRN12620001003965 | An RCT of a decision aid to support informed choices about taking aspirin to prevent colorectal cancer and other chronic diseases: a study protocol for the SITA (Should I Take Aspirin?) trial | <https://www.anzctr.org.au/Trial/Registration/TrialReview.aspx?ACTRN=12620001003965> | RCT | NR  Expected completion date: NR | 258 participants | *Multi-component*: educational meetings (video) and materials (videos, brochures)  vs. no intervention | Adherence to medication | No | Australia,  outpatient |

NR= not reported; RCT= randomized controlled trial; QoL= Quality of life.

## **Additional File 7. Characteristics of studies awaiting classification**

| Study  ID | Title | Links | Design | Status | Nr. of participants/ patients/ clusters | Intervention & comparator | Outcomes | Results available | Country,  Setting |
| --- | --- | --- | --- | --- | --- | --- | --- | --- | --- |
| NCT03008993 | Effectiveness of the HuCare Quality Improvement Strategy on health-related quality of life in patients with cancer: study protocol of a stepped-wedge cluster randomized controlled trial (HuCare2 study) | <https://pubmed.ncbi.nlm.nih.gov/28988170/>  <https://clinicaltrials.gov/ct2/show/NCT03008993?term=NCT03008993&draw=2&rank=1> | Stepped-wedge  cluster RCT | Status: *completed*  But no published results; Authors contacted, response | 762 patients within 3 clusters | *Multi-component*:  Hucare Quality Improvement Strategy - HQIS in 3 phases:  (1) clinician training (educational session) (2) center support - 4 on site visits by experts of the project team (audit and feedback) (3) implementation of 6 EbM recommendations  vs. no intervention | QoL; Clinical staff knowledge | No | Italy,  inpatient |
| NCT02046811 | Advancing Survivors’ Knowledge (ASK) about skin cancer study: study protocol for a randomized controlled trial | <https://clinicaltrials.gov/ct2/show/NCT02046811?term=NCT02046811&draw=2&rank=1>  <https://www.ncbi.nlm.nih.gov/pmc/articles/PMC4392639/> | RCT | Status: *completed*  But no published results; Authors contacted, no response yet | 726 patients | *Multi-component*:  (1) Patient  Activation and Education (PAE), (2) PAE plus physician activation (PAE +MD) adding physician activation/educational materials and, (3) PAE plus physician activation, plus teledermoscopy (PAE + MD + TD)  (EPOC: educational meetings and materials, patient-mediated intervention, The use of information and communication technology) vs. interventions serve as comparators | Physician skin examination prior to 18-month survey; Reduction of the time  interval between the first finding of a suspect lesion and a diagnostic visit etc. | No | USA,  inpatient |
| NCT03291587 | The OaSiS trial: A hybrid type II, national cluster randomized trial to implement smoking cessation during CT screening for lung cancer | <https://www.sciencedirect.com/science/article/pii/S1551714420300410?via%3Dihub>  <https://clinicaltrials.gov/ct2/show/study/NCT03291587?term=NCT03291587&draw=2&rank=1> | Cluster  RCT | Status: *completed*  But no published results; Authors contacted, response | 1053 patients, 60 healthcare professionals | *Multi-component*:  (1) Webinars, (2) Site Visit and Strategic Planning, (3) Performance Coaching using Audit and Feedback, and (5) Peer Learning Calls (EPOC: educational meetings and materials, audit and feedback, educational outreach visits, use of information and communication technology) vs.  No intervention (usual care) | Proportion of Patients with 7-day Smoking Abstinence; Salivary Cotinine Test for Non-Smokers etc | No | USA,  inpatient |
| Not reported | Abstract title: *Cancer Pain: Bridging the Evidence-practice Gap*, Abstract Nr: P91 | <https://journals.sagepub.com/doi/pdf/10.1177/0269216316646056> | Stepped-wedge  cluster RCT | Conference abstract;  Authors contacted, no response yet | 492 patients | *Multi-component*:  (1) patient goal setting tool, pain management plan and diary, (2) QStream health professional education program;  (3) audit and feedback regarding centre performance  on key standards of care  (EPOC: educational meetings and materials, audit and feedback) | QoL; Pain severity;  Carer experience; Cost effectiveness | No | Australia,  outpatient |
| Not reported | Abstract title: *Implementation strategies for cancer pain management guidelines*, Abstract Nr. P088 | [EAPC2016: Abstracts, 2016 (sagepub.com)](https://journals.sagepub.com/doi/10.1177/0269216316646056) | Cluster RCT | Conference abstract | Not reported | *Multi-component*:  (1) patient self-management resources, (2) QStream health professional education program;  (3) audit and feedback (EPOC: educational meetings and materials, audit and feedback) | QoL; Pain severity;  Carer experience; Cost effectiveness | No | Australia,  outpatient |
| Not reported | Abstract title: *A cluster randomised controlled trial of guidelines and screening with implementation strategies versus guidelines and screening alone to improve pain in adults with cancer*, Abstract Nr. CANCE-03 | [Abstracts for MASCC/ISOO Annual Meeting 2021 \| SpringerLink](https://link.springer.com/article/10.1007/s00520-021-06285-8) | Stepped-wedged cluster RCT | Conference abstract | 754 patients | Not clearly reported (“tailored implementation strategies”) | Pain, QoL, carer experience, cost-effectiveness | No | Australia,  outpatient |
| Not reported | Abstract title: Using the Oncology Care Model to Manage Cancer Pain at an Outpatient Oncology Clinic | <https://store.ons.org/cjon/26/1/using-oncology-care-model-manage-cancer-pain-outpatient-oncology-clinic> | Pre-post study | Only abstract available | Not reported | Not clearly reported in the abstract | Cancer pain, patient satisfaction | Un-available full text | Not reported, outpatient |

NR= not reported; RCT= randomized controlled trial; QoL= Quality of life.

## **Additional File 8. Risk of bias judgement for randomized controlled trials**

| **Study** | **Risk of bias domain** | | **Judgement** | **Support for judgement** |
| --- | --- | --- | --- | --- |
| **Brown 2018** | Random sequence generation  (**selection bias**) | | Low RoB | *Quote*: “A stepped-wedge cluster randomized design was used. Participating Sites crossed over from the pre- to post-intervention phase in nine randomized steps, determined by a computer-generated random number sequence, with the intervention rolled out during regularly scheduled MDT meetings”  *Comment*: method of randomization described |
|  | Allocation concealment (**selection bias**) | | Unclear RoB | *Comment*: Not described in sufficient detail to assess RoB. |
|  | Blinding participants and personnel (**performance bias**) | | High RoB | *Comment*: Only the independent research assistants, that collected the data, were blinded. Participants (urologists) and other study staff were probably not blinded. |
|  | Blinding outcome assessment  (**detection bias**) | **Objective outcomes**:  Referral (Proportion of patients referred to RP) | Low RoB | *Quote*: “Clinical data were extracted by independent research assistants, blinded to the date of intervention commencement, from medical records at hospitals, cancer centers and urologists’ private consulting rooms, for a minimum of 6 months after RP, using standard methods. Data were collected for all patients who had a RP performed by a participating urologist.”  *Comment*: blinded independent research assistants |
|  |  | **Subjective outcomes**:  Attitudes and knowledge | High RoB | *Comment*: Unclear whether subjective outcomes (attitudes and knowledge) measured by survey may have been influenced as no information regarding blinding participants was reported. For subjective outcomes unclear when attitudes were measured (follow-up period not clearly reported). Participants were most likely not blinded. |
|  | Incomplete outcome data (**attrition bias**) | **Objective outcomes**:  Referral (Proportion of patients referred to RP) | Low RoB | *Comment*: Patient loss in every group (Control, Transition, Intervention) below 2%. |
|  |  | **Subjective outcomes**:  Attitudes and knowledge | Unclear RoB | *Comment*: Only 54% of all urologists completed both intervention surveys (pre and post). |
|  | Selective reporting (**reporting bias**) | | Low RoB | *Comment*: Selective outcome reporting bias not detected, trial protocol and ClinicalTrials.gov record assessed. |
|  | Other sources of bias (e.g. for cluster RCTs: recruitment bias, loss of clusters, incorrect analysis) | | Unclear RoB | *Quote*: “While there was 100% participation at five of nine Sites, not all eligible urologists participated at all sites which may have resulted in volunteer bias.”  *Comment*: There is insufficient rationale or evidence that the identified problem has introduced bias. |
|  | **Overall Rob** | **Objective outcomes**:  Referral | High RoB | At least one domain with high RoB |
|  |  | **Subjective outcomes**:  Attitudes and knowledge | High RoB | At least one domain with high RoB |
| **McCarter 2018** | Random sequence generation  (**selection bias**) | | Low RoB | *Quote*: “The EAT trial used a stepped-wedge cluster randomized design. The order in which the intervention was introduced to radiotherapy departments was randomly allocated by an independent statistician using a uniform random number generator in Stata (StataCorp, College Station, TX)” *Comment*: method of randomization described |
|  | Allocation concealment (**selection bias**) | | Unclear RoB | *Comment*: Not described in sufficient detail to assess RoB. |
|  | Blinding participants and personnel (**performance bias**) | | High RoB | *Quote*: “Site staff (dieticians, data managers) were not blind to participant allocation. However, patients were blind to condition.”  *Comment*: Performance bias due to knowledge of the allocated interventions by participants and personnel during the study. |
|  | Blinding outcome assessment (**detection bias**) | **Objective outcomes**:  Screening & referral (*depression screening, depression referral*) | High RoB | *Quote*: “Site staff (dieticians, data managers) were not blind to participant allocation, which may have introduced bias into dietician documentation of the provision of guideline recommendations and data manager chart reviews of patient medical records”  *Comment*: Detection bias due to knowledge of the allocated interventions by outcome assessors. For subjective outcomes unclear when attitudes were measured (follow-up period not clearly reported). |
|  |  | **Subjective outcomes**:  Attitudes (*Helpfulness of practice change strategies*) | High RoB |  |
|  | Incomplete outcome data (**attrition bias**) | | Low RoB | *Quote*: “…intention to treat framework, using six logistic regression models, including fixed effects for study stage (intervention or control phase) and study site (hospital).”  *Comment*: Handling of incomplete outcome data was complete and unlikely to have produced bias. |
|  | Selective reporting (**reporting bias**) | | Low RoB | *Comment*: Selective outcome reporting bias not detected, trial protocol and ClinicalTrials.gov record assessed (not all outcomes reported in this trial, because it is only a part of the EAT trial) |
|  | Other sources of bias (e.g. for cluster RCTs: recruitment bias, loss of clusters, baseline imbalance, incorrect analysis) | | Low RoB | *Comment*: No other sources of bias detected |
|  | **Overall RoB** | **Objective outcomes**:  Screening & referral | High RoB | At least one domain with high RoB |
|  |  | **Subjective outcomes**:  Attitudes | High RoB | At least one domain with high RoB |
| **Gilbert 2021** | Random sequence generation  (**selection bias**) | | Low RoB | *Quote*: “an open-label prospective multicenter cluster-randomized trial with a stepped-wedge design. All centers (clusters) started by including patients in the control phase (management as usual), and the implementation of the intervention was rolled out sequentially in each center every six months until all centers benefited from the intervention phase for at least six months. The time at which each cluster switched from the control condition to intervention was randomized.” |
|  | Allocation concealment (**selection bias**) | | Unclear RoB | *Comment*: Not described in sufficient detail to assess RoB. |
|  | Blinding participants and personnel (**performance bias**) | | High RoB | *Comment*: Open-label RCT. Performance bias due to knowledge of the allocated interventions by participants and personnel during the study. |
|  | Blinding outcome assessment (**detection bias**) | **Objective outcomes**:  Overall survival, Adverse events, Screening | High RoB | *Quote*: “Data were collected for both groups from medical records by a clinical research  assistant and reported in an electronic Case Report Form (CRF).”  *Comment*: Detection bias possible due the lack of blinding of outcome assessors. |
|  | Incomplete outcome data (**attrition bias**) | | Low RoB | *Quote*: “Finally, we considered 147 patients for the intention-to-treat analysis, among which 74 patients were in the intervention condition and 73 were in the control condition.”  *Comment*: Outcome data was complete and handling is unlikely to have produced bias. |
|  | Selective reporting (**reporting bias**) | | Low RoB | *Comment*: Selective outcome reporting bias not detected, trial protocol and registration ([NCT02084524](https://clinicaltrials.gov/show/NCT02084524)) available. |
|  | Other sources of bias (e.g. for cluster RCTs: recruitment bias, loss of clusters, baseline imbalance, incorrect analysis) | | Low RoB | *Comment*: No other sources of bias detected |
|  | **Overall RoB** | **Objective outcomes**:  Adverse events, Screening, Prescribing behaviour | High RoB | At least one domain with high RoB |
| **Lovell 2022** | Random sequence generation  (**selection bias**) | | Low RoB | *Quote*: “A stepped wedge, cluster-randomized trial approach was taken in which clusters were randomized to commence the intervention at different times following an initial control period in which outcomes were measured for usual care. A training phase enabled the transition from control to intervention, during which recruitment and measurement were placed on hold. The order in which each center moved from control to intervention phase was randomly allocated by a computer algorithm performed by the study statistician.” *Comment*: method of randomization described |
|  | Allocation concealment (**selection bias**) | | High RoB | *Quote*: “Allocation of clusters could not be concealed from clinicians and managers.” |
|  | Blinding participants and personnel (**performance bias**) | | High RoB | *Quote*: “Blinding for center staff and the project team collecting data was not possible. However, information for patients provided only general information about the aims of the study, not the specifics of the design and intervention. Patients were allocated based on whether the center they attended was in the control or intervention phase at the time they were first screened as having worst pain rated 2 or more on the NRS.”  *Comment*: Performance bias due to knowledge of the allocated interventions by participants and personnel during the study. |
|  | Blinding outcome assessment (**detection bias**) | **Subjective outcomes**:  Quality of life | High RoB | *Quote*: “Blinding for center staff and the project team collecting data was not possible."  *Comment*: Detection bias due to knowledge of the allocated interventions by outcome assessors. |
|  | Incomplete outcome data (**attrition bias**) | | Low RoB | *Comment*: Outcome data was complete and handling is unlikely to have produced bias. |
|  | Selective reporting (**reporting bias**) | | Low RoB | *Comment*: Selective outcome reporting bias not detected, trial protocol and trial registration assessed. |
|  | Other sources of bias (e.g. for cluster RCTs: recruitment bias, loss of clusters, baseline imbalance, incorrect analysis) | | Low RoB | *Comment*: No other sources of bias detected |
|  |  | **Subjective outcomes**:  Quality of life | High RoB | At least one domain with high RoB |
| **Mohile 2021** | Random sequence generation  (**selection bias**) | | Low RoB | *Quote*: “Practice clusters were randomly assigned (1:1) to one of the two study groups (the geriatric assessment intervention or standard of care) by means of a computer generated randomisation table. The randomisation was stratified by practice size.”  *Comment*: method of randomization described |
|  | Allocation concealment (**selection bias**) | | Unclear RoB | *Comment*: Not described in sufficient detail to assess RoB. |
|  | Blinding participants and personnel (**performance bias**) | | High RoB | *Quote*: “Because this study evaluated a model of care, participants and staff at the community oncology clinics were not masked. Other than the statisticians who completed the analyses, all Research Base investigators were masked to the assignment. Furthermore, masking was preserved among the clinical team members who centrally reviewed treatment and toxic effect data.”  *Comment*: Performance bias due to knowledge of the allocated interventions by participants. |
|  | Blinding outcome assessment (**detection bias**) | **Objective outcomes**:  Overall survival, Adverse events, Prescribing behaviour | Low RoB | *Quote*: “.. all Research Base investigators were masked to the assignment. Furthermore, masking was preserved among the clinical team members who centrally reviewed treatment and toxic effect data. Masked oncology clinicians reviewed medical records to verify.” |
|  | Incomplete outcome data (**attrition bias**) | | Low RoB | *Comment*: Outcome data was complete and handling is unlikely to have produced bias. |
|  | Selective reporting (**reporting bias**) | | Low RoB | *Comment*: Selective outcome reporting bias not detected, trial protocol and registration available. |
|  | Other sources of bias (e.g. for cluster RCTs: recruitment bias, loss of clusters, baseline imbalance, incorrect analysis) | | Low RoB | *Comment*: No other sources of bias detected |
|  | **Overall RoB** | **Objective outcomes**:  Overall survival, Adverse events, Prescribing behaviour | High RoB | At least one domain with high RoB |

RoB= Risk of Bias

## **Additional File 9. Risk of bias judgement for non-randomized controlled studies of interventions**

| **Study** | **Risk of bias domain** | | **Judgement** | **Support for judgement** |
| --- | --- | --- | --- | --- |
| **Bonkow-ski 2018** | Bias due to confounding | | Serious RoB | *Comment*: Confounding cannot be excluded, still, relevant differences regarding age, gender, co-morbidities, work experience between groups were measured. |
|  | Bias in selection of participants into the study | | Moderate RoB | *Comment*: Selection into the study may have been related to intervention and outcome  *and*  Methods of selection and concealment were not sufficiently described. |
|  | Bias in classification of interventions | | Low RoB | *Comment*: Intervention is well defined;  *and*  Intervention definition is based solely on information collected at the time of intervention |
|  | Bias due to deviations from intended interventions (Effect of assignment to intervention) | | Serious RoB | *Quote*: “Providers that knew of the initiative began to discontinue IV narcotic medications prior to discharge”. |
|  | Bias due to missing data | | Low RoB | *Comment*: Proportions of and reasons for missing participants were similar across intervention groups (flow-diagram). |
|  | Bias in measurement of outcomes | **Objective outcomes** Prescribing behaviour (*narcotic administration 24 before discharge)* | Serious RoB | *Quote*: “Neither the practice or attitudes surveys were tested for reliability or validity. Providers that knew of the initiative began to discontinue IV narcotic medications prior to discharge. It makes it challenging to determine whether this is the reason fewer IV narcotics were administered prior to discharge or whether the education and guideline led to less use by nurses.”  *Comment*: The outcome was assessed by assessors aware of the intervention and this may have influenced the outcome. |
|  | Bias in selection of the reported result | | Moderate RoB | *Comment*: No registered protocol. The outcome measurements and analyses are consistent with the methods and are clearly defined *and*  (ii) There is no indication of selection of the reported analysis from among multiple analyses; *and*  (iii) There is no indication of selection of the cohort or subgroups for analysis and reporting based on the results. |
|  | **Overall RoB (objective outcomes)** | | Serious RoB | *Quote*: The study is judged to be at serious risk of bias in at least one domain, but not at critical risk of bias in any domain. |

| **Bonkow-ski 2018** | Bias due to confounding | | Serious RoB | *Comment*: Confounding cannot be excluded, still, relevant differences regarding age, gender, co-morbidities, work experience between groups were measured. |
| --- | --- | --- | --- | --- |
|  | Bias in selection of participants into the study | | Moderate RoB | *Comment*: Selection into the study may have been related to intervention and outcome  *and*  This could not be adjusted for in analyses. |
|  | Bias in classification of interventions | | Low RoB | *Comment*: Intervention is well defined;  *and*  Intervention definition is based solely on information collected at the time of intervention |
|  | Bias due to deviations from intended interventions (Effect of assignment to intervention) | | Low RoB | *Comment*: Any deviations from usual practice were unlikely to impact on the outcome. No deviations from intended intervention detected. |
|  | Bias due to missing data | | Low RoB | *Comment*: Proportions of and reasons for missing participants were similar across intervention groups (flow-diagram). |
|  | Bias in measurement of outcomes | **Subjective outcomes** Prescribing behaviour (*changes in nursing pain management*); Attitudes | Serious RoB | *Quote*: “Neither the practice or attitudes surveys were tested for reliability or validity.”  *Comment*: The outcome measure was subjective (i.e. vulnerable to influence by the knowledge of the intervention received by study participants);  *and*  The outcome was assessed by assessors aware of the intervention. |
|  | Bias in selection of the reported result | | Serious RoB | *Comment*: No registered protocol found. Nurses satisfaction with the intervention was poorly reported, only selected items were described. |
|  | **Overall RoB (subjective outcomes)** | | Serious RoB | *Quote*: The study is judged to be at serious risk of bias in at least one domain, but not at critical risk of bias in any domain. |

| **Cowperth-waite 2019** | Bias due to confounding | | Serious RoB | *Quote*: „ Neither the patient nor the RN groups were perfectly similar in the pre- and post-intervention samples, possibly affecting results. Patients in the post-intervention group were less likely to have been receiving chemotherapy within the past 30 days. There were differences between the pre- and post-intervention RNs in terms of attendance at end of life training and mean years of experience.”  *Comment*: Confounding cannot be excluded, still, relevant differences regarding age, gender, co-morbidities, work experience between groups were measured and discussed. |
| --- | --- | --- | --- | --- |
|  | Bias in selection of participants into the study | | Low RoB | *Quote*: “Patients admitted to the solid tumour medical oncology unit who had a recorded pain intensity score of 1 or greater at any time during hospitalization were included in the study. HCAHPS surveys were mailed to a random sample of discharged patients through Press Ganey, a patient experience consulting company. Survey results were accessed from the Press Ganey website for each of the pre- and post-intervention time periods. The unit’s RNs received email invitations to complete the KAP Electronically.  *Comment*: All participants who would have been eligible for the target trial were included in the study; *and* For each participant, the start of follow-up and the start of intervention coincided. |
|  | Bias in classification of interventions | | Low RoB | *Comment*: Intervention is well defined;  *and*  Intervention definition is based solely on information collected at the time of intervention |
|  | Bias due to deviations from intended interventions (Effect of assignment to intervention) | | Low RoB | *Comment*: Any deviations from usual practice were unlikely to impact on the outcome. |
|  | Bias due to missing data | | Low RoB | *Comment*: Proportions of and reasons for missing participants were similar across intervention groups. |
|  | Bias in measurement of outcomes | **Subjective outcomes**: Quality of life (*Pain intensity*); Knowledge and attitudes | Serious RoB | *Quote*: “The KAP survey was altered from the original, possibly affecting validity and reliability.  *Comment*: The outcome measure was subjective (i.e. vulnerable to influence by the knowledge of the intervention received by study participants); Follow-up periods were not reported.  *and*  The outcome was assessed by assessors aware of the intervention. |
|  | Bias in selection of the reported result | | Serious RoB | *Comment*: No registered protocol found. The outcome measurements and analyses are consistent with the methods. Still, the measurement of attitudes and knowledge are unclearly reported. Only 2 question items were reported that could be intentionally selected. |
|  | **Overall RoB (subjective outcomes)** | | Serious RoB | *Quote*: The study is judged to be at serious risk of bias in at least one domain, but not at critical risk of bias in any domain. |

| **Knoerl 2021** | Bias due to confounding | | Serious RoB | *Comment*: A relevant confounder-work experience was not measured. Also the group was rather heterogenous due to many clinical specialities, which may have lowered the comparability between the participants. |
| --- | --- | --- | --- | --- |
|  | Bias in selection of participants into the study | | Serious RoB | *Comment*: Although the intervention and the clinicians stayed the same throughout the study, the individual patient eligibility criteria were amended during the trial to enhance recruitment. This has introduced changes in CIPN severity over time (e.g., different types, dosages and time points of neurotoxic chemotherapy -thus, for pre- and post-intervention measurements). |
|  | Bias in classification of interventions | | Low RoB | *Comment*: Intervention is well defined;  *and*  Intervention definition is based solely on information collected at the time of intervention |
|  | Bias due to deviations from intended interventions (Effect of assignment to intervention) | | Low RoB | *Comment*: Any deviations from usual practice were unlikely to impact on the outcome. No deviations from intended intervention detected. |
|  | Bias due to missing data | | Low RoB | *Comment*: Proportions of and reasons for missing participants were similar across intervention groups (flow-diagram). |
|  | Bias in measurement of outcomes | **Objective outcomes**: Prescribing behaviour (*frequency of appropriate CIPN management)* | Moderate RoB | *Quote*: “Study staff abstracted clinicians’ documentation (yes/no) of CIPN assessment and management. All medical record abstraction was conducted by the principal investigator and two study staff members. All identified discrepancies were resolved between the principal investigator and study staff. It is possible that the methods used to measure changes in clinicians’ CIPN assessment and management documentation may not have been sensitive to the unique symptom presentations associated with CIPN due to taxanes, oxaliplatin, or bortezomib.“  *Comment*: The methods of outcome assessment were comparable across intervention groups; *and*  (ii) The outcome measure is only minimally influenced by the knowledge of the intervention received by study participants. Still, follow-up periods not clearly described. |
|  | Bias in selection of the reported result | | Moderate RoB | *Comment*: No registered protocol found. The outcome measurements and analyses are consistent with the methods and are clearly defined *and*  (ii) There is no indication of selection of the reported analysis from among multiple analyses; *and*  (iii) There is no indication of selection of the cohort or subgroups for analysis and reporting based on the results. |
|  | **Overall RoB (objective outcomes)** | | Serious RoB | *Quote*: The study is judged to be at serious risk of bias in at least one domain, but not at critical risk of bias in any domain. |

| **Knoerl 2021** | Bias due to confounding | | Serious RoB | *Quote*: “Between-group changes in patients’ CIPN severity over  time was confounded by the eligibility criteria (e.g., patients were receiving various neurotoxic chemotherapy  types/dosages and recruited at different time points during their neurotoxic chemotherapy regimens).”  *Comment*: Time-varying confounding cannot be excluded. |
| --- | --- | --- | --- | --- |
|  | Bias in selection of participants into the study | | Serious RoB | *Comment*: Although the intervention and the clinicians stayed the same throughout the study, the individual patient eligibility criteria were amended during the trial to enhance recruitment. This has introduced changes in CIPN severity over time (e.g., different types, dosages and time points of neurotoxic chemotherapy -thus, for pre- and post-intervention measurements). |
|  | Bias in classification of interventions | | Low RoB | *Comment*: Intervention is well defined;  *and*  Intervention definition is based solely on information collected at the time of intervention |
|  | Bias due to deviations from intended interventions (Effect of assignment to intervention) | | Low RoB | *Comment*: Any deviations from usual practice were unlikely to impact on the outcome. No deviations from intended intervention detected. |
|  | Bias due to missing data | | Low RoB | *Comment*: Proportions of and reasons for missing participants were similar across intervention groups (flow-diagram). |
|  | Bias in measurement of outcomes | **Subjective outcomes**: Quality of life (*sensory and motor CIPN severity, worst pain intensity*);  Attitudes (*acceptability and feasibility*) | Serious RoB | *Comment*: The outcome measures were subjective (i.e. vulnerable to influence by knowledge of the intervention received by study participants); Follow-up periods were not clearly reported.  *and*  The outcome was assessed by assessors most probably aware of the intervention. |
|  | Bias in selection of the reported result | | Moderate RoB | *Comment*: No registered protocol found. The outcome measurements and analyses are consistent with the methods and are clearly defined *and*  (ii) There is no indication of selection of the reported analysis from among multiple analyses; *and*  (iii) There is no indication of selection of the cohort or subgroups for analysis and reporting based on the results. |
|  | **Overall RoB (subjective outcomes)** | | Serious RoB | *Quote*: The study is judged to be at serious risk of bias in at least one domain, but not at critical risk of bias in any domain. |

| **Phillips 2017** | Bias due to confounding | | Critical RoB | *Quotes: “*Our inability to control for confounders and participant bias is a major limitation of this pilot study. It is possible that the participants who completed the intervention differed from the nonparticipants in terms of their motivations and capabilities.” Patients included after audit were younger than patients included in first assessment.”  *Comment*: Confounding inherently not controllable. Time-varying confounding cannot be excluded and it was not adjusted and not controlled for. |
| --- | --- | --- | --- | --- |
|  | Bias in selection of participants into the study | | Moderate RoB | *Comment*: Possible volunteer bias due to self-selection intro the study, younger vs. older patients- imbalances); and This could not be adjusted for in analyses (Took younger participants in T3)- not enough data reported to judge if this caused bias. |
|  | Bias in classification of interventions | | Low RoB | *Comment*: Intervention status is well defined;  *and*  Intervention definition is based solely on information collected at the time of intervention |
|  | Bias due to deviations from intended interventions (Effect of assignment to intervention) | | Low RoB | *Comment*: Any deviations from usual practice were unlikely to impact on the outcome. No deviations from intended intervention detected. |
|  | Bias due to missing data | | Serious RoB | *Quote*: “Despite the high attrition between the T1 survey and enrolment into the online pain assessment learning module, participant engagement was high once the module commenced, with a 90% completion rate. It is possible that the participants who completed the intervention differed from the nonparticipants in terms of their motivations and capabilities.”-  *Comment*: Proportions of missing participants differ substantially across interventions. |
|  | Bias in measurement of outcomes | **Objective outcomes**: Knowledge (*Pain assessment chart audit changes*) | Moderate RoB | *Quote*: “All chart audit abstractions were undertaken by a trained research assistant using a case report form supported by a data dictionary. This process ensured standardized audit data collection at all sites.”  *Comment*: Chart audits were undertaken by an unblinded research assistant, but still standardised. |
|  | Bias in selection of the reported result | | Serious RoB | *Comment*: No registered protocol found. The outcome measurements and analyses are consistent with the methods. Still, objective knowledge (pain assessment chart audit changes) measurement was not clearly defined (whether mean or medians were used). |
|  | **Overall RoB (objective outcomes)** | | Critical RoB | *Quote*: The study is judged to be at critical risk of bias in at least one domain. |

| **Phillips 2017** | Bias due to confounding | | Critical RoB | *Comment*: potential confounders like age, gender, work experience and knowledge etc. not controlled for |
| --- | --- | --- | --- | --- |
|  | Bias in selection of participants into the study | | Moderate RoB | *Comment*: Possible volunteer bias due to self-selection intro the study, younger vs. older patients- imbalances); and This could not be adjusted for in analyses (Took younger participants in T3)- not enough data reported to judge if this caused bias. |
|  | Bias in classification of interventions | | Low RoB | *Comment*: Intervention status is well defined;  *and*  Intervention definition is based solely on information collected at the time of intervention |
|  | Bias due to deviations from intended interventions (Effect of assignment to intervention) | | Low RoB | *Comment*: Any deviations from usual practice were unlikely to impact on the outcome. No deviations from intended intervention detected. |
|  | Bias due to missing data | | Serious RoB | *Quote*: “Despite the high attrition between the T1 survey and enrolment into the online pain assessment learning module, participant engagement was high once the module commenced, with a 90% completion rate. It is possible that the participants who completed the intervention differed from the nonparticipants in terms of their motivations and capabilities.”-  *Comment*: Proportions of missing participants differ substantially across interventions. |
|  | Bias in measurement of outcomes | **Subjective outcomes**: Knowledge (*Perceived knowledge*; *assessment tool*) Attitudes (*Confidence)* | Serious RoB | *Quote*: “To minimize bias, the chart audit period inclusion dates were blinded to all participants and manager (…) The observed changes in nurses’ pain assessment capabilities and cancer pain assessment practices may be due to the online education intervention alone and/or awareness that the charts would be audited (…)”  *Comment*: The outcome measure was subjective (i.e. vulnerable to influence by the knowledge of the intervention received by study participants);  *and*  The outcome was assessed by assessors aware of the intervention received by study participants (self-rated outcomes by study participants) |
|  | Bias in selection of the reported result | | Moderate RoB | *Comment*: No registered protocol found. The outcome measurements and analyses are consistent with the methods and are clearly defined *and*  (ii) There is no indication of selection of the reported analysis from among multiple analyses; *and*  (iii) There is no indication of selection of the cohort or subgroups for analysis and reporting based on the results. |
|  | **Overall RoB (subjective outcomes)** | | Critical RoB | *Quote*: The study is judged to be at critical risk of bias in at least one domain. |

RoB= Risk of Bias

## **Additional File 10. Risk of bias summary plots**

**Risk of bias summary plot for randomized controlled trials**

***
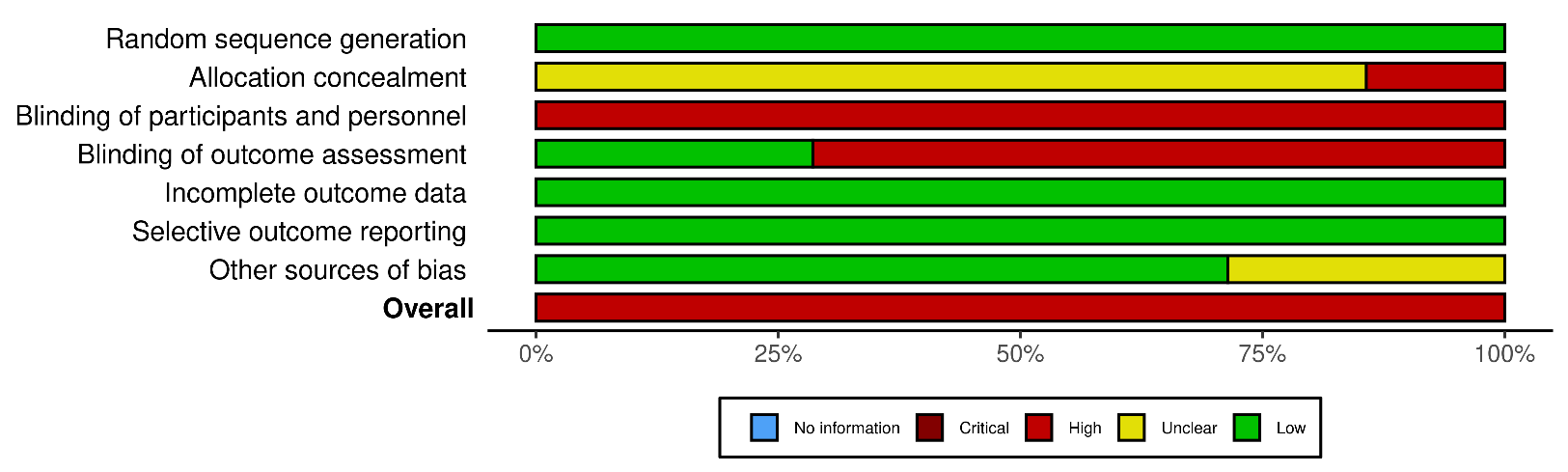
***

**Risk of bias summary plot for non-randomized studies of interventions**

**
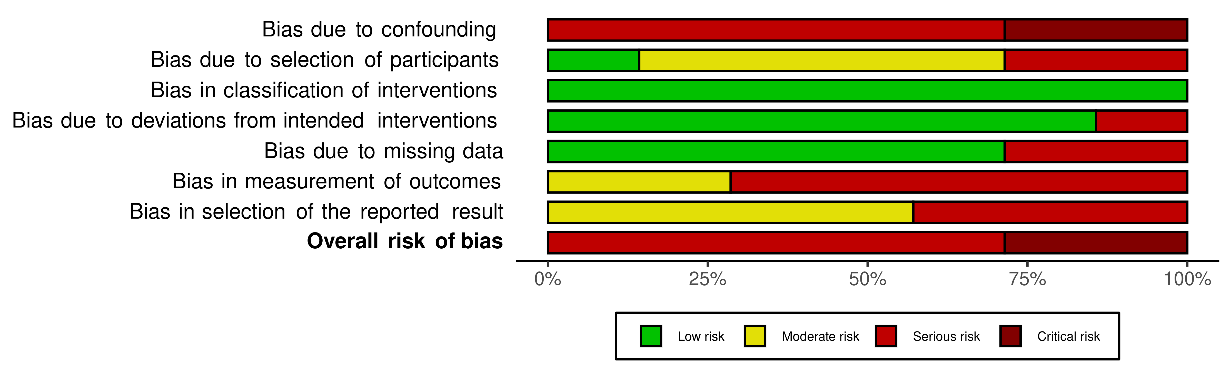
**

## **Additional File 11. Outcome effect tables**

**eTable 1. Overall survival**

| Overall survival | | | | | | | | | | | |
| --- | --- | --- | --- | --- | --- | --- | --- | --- | --- | --- | --- |
| Study | **Definition of outcome** | **Outcome measurement** | **Follow-up** | **I**  **(Event)** | **I**  **(N)** | **C**  **(Event)** | **C**  **(N)** | **Effect measure** | **between-group difference** | **Measure of precision for between group difference** | **Risk of bias** |
| Gilbert 2021 | Survival 30 days post-surgery | NR | 30 days | 70** | 74 | 73** | 73 | RR* | 0.946 | 95% CI:  0.769 to 1.228,  p = 0.813 | High |
| Mohile 2021 | Patients alive at 6 months | Hazard ratio | 6 months | 250 (72%) | 349 | 275 (75%) | 369 | Adjusted HR | 1.13 | 95% CI:  0.85 to 1.50,  p = 0.68 | High |
|  | Survival over 1 year | Hazard ratio | 1 year | NR | 349 | NR | 369 | Adjusted HR | 1.05 | 95% CI:  0.85 to 1.29,  p= 0.68 |  |

N= number of patients analyzed; I= intervention arm; C= control arm; HR= Hazard ratio; *= self-calculated; **= survival as event.

**eTable 2. Quality of life**

| Quality of life | | | | | | | | | | | |
| --- | --- | --- | --- | --- | --- | --- | --- | --- | --- | --- | --- |
| Study | **Definition of**  **outcome** | **Outcome measurement** | **Follow-up** | **I**  **Mean (SD)** | **I**  **(N)** | **C**  **Mean (SD)** | **C**  **(N)** | **Effect measure** | **between-group difference** | **Measure of precision for between group difference** | **Risk of bias** |
| Cowperth-waite  2019 | Pain intensity | Scores ranged from 0 (no pain) to 10  (worst pain imaginable) | T1  (First) | 3.23  (3.31) | 157 | 3.14 (3.18) | 173 | Mean difference* | 0.090 | 95% CI:  -0.6131 to 0.7931,  p= 0.8013 | Serious |
|  |  |  | T2  (Last) | 2  (2.67) | 157 | 1.79 (2.48) | 173 | Mean difference* | 0.210 | 95% CI:  -0.3477 to 0.7677,  p= 0.4594 |  |
| Lovell 2022 | Pain score reduction of 30% among those with ≥5 NRS on worst pain at week 1 | Pain Numeric Rating Scale (NRS) with NRS ≥ 2 as clinically relevant or NRS ≥5 as moderate to severe pain | At week 1 | 30 (11.8) | 264 | 31 (11.9) | 280 | OR | 1.12 | 95% CI:  0.79 to 1.60,  p= 0.51 | High |
|  | Total EORTC QLQ-C15-PAL score | Score Range: 1 “not at all”, 2 “a little”, 3 “quite a bit” and 4 “very much” | At 1 week | 14.6 (1.8) | 264 | 14.6 (1.9) | 280 | Mean difference* | 0.00 | 95% CI:  -0.312 to 0.312,  p= 1.000 |  |
|  |  |  | At 2 weeks | 14.9 (1.8) | 264 | 14.5 (2.2) | 280 | Mean difference* | -0.40 | 95% CI:  -0.739 to -0.060,  p= 0.02 |  |
|  |  |  | At 4 weeks | 14.7 (1.8) | 264 | 14.8 (1.9) | 280 | Mean difference* | 0.10 | 95% CI:  -0.212 to 0.412,  p= 0.629 |  |
|  | Worst pain NRS score | Numeric rating scale (NRS)  0–10 scale, with zero meaning “no pain” and 10 meaning “the worst pain imaginable” | At 1 week | 4.3 (2.3) | 264 | 4.4 (2.4) | 280 | Mean difference* | 0.10 | 95% CI:  -0.296 to 0.931,  p= 0.496 |  |
|  |  |  | At 2 weeks | 4.2 (2.4) | 264 | 4.7 (2.7) | 280 | Mean difference* | 0.50 | 95% CI:  0.068 to 0.496,  p= 0.023 |  |
|  |  |  | At 4 weeks | 4.3 (2.7) | 264 | 4.4 (2.5) | 280 | Mean difference* | 0.10 | 95% CI:  -0.338 to 0.538,  p= 0.654 |  |
| Knoerl 2021 | Sensory CIPN Severity | Changes in CIPN Patient-Reported on  EORTC QLQ-CIPN20 Scale- contains 20 items and uses a 4-point Lickert scale (1 = “not at all,” 2 = “a little,” 3 = “quite a bit,” and 4 = “very much”)- higher scores indicate more symptom burden | T1 | 7.48  (9.2) | 71 | 8.21 (11.77) | 69 | Mean difference* | 0.730 | 95% CI:  -2.7947 to 4.2547,  p= 0.6828 | Serious |
|  |  |  | T2 | 9.47  (8.74) | 71 | 12.62  (12.83) | 69 | Mean difference* | 3.150 | 95% CI:  -0.5095 to 6.8095,  p= 0.0910 |  |
|  |  |  | T3 | 11.21 (11.45) | 72 | 12.68  (12.38) | 66 | Mean difference* | 1.470 | 95% CI:  -2.5415 to 5.4815,  p= 0.4699 |  |
|  | Motor CIPN Severity |  | T1 | 4.93  (6.42) | 71 | 5.38 (8.7) | 69 | Mean difference* | 0.450 | 95% CI:  -2.1001 to 3.0001,  p= 0.7277 |  |
|  |  |  | T2 | 7.89  (8.91) | 70 | 7.32 (10.57) | 69 | Mean difference* | -0.570 | 95% CI  -3.8472 to 2.7072,  p= 0.7314 |  |
|  |  |  | T3 | 8.04  (9.33) | 72 | 8.98 (11.84) | 66 | Mean difference* | 0.940 | 95% CI  -2.6336 to 4.5136,  p= 0.6038 |  |
|  | Worst CIPN Pain Intensity | Changes in CIPN Patient-Reported-Worst CIPN Numerical Rating Scale- ranges from 0-10, higher scores on the NRS indicate more severe pain intensity | T1 | 1.43  (1.79) | 69 | 1.38 (2.17) | 69 | Mean difference* | -0.050 | 95% CI.  -0.7197 to 0.6197,  p= 0.8828 |  |
|  |  |  | T2 | 1.97  (2.3) | 72 | 2.01 (2.54) | 68 | Mean difference* | 0.040 | 95% CI:  -0.7721 to 0.8521,  p= 0.9226 |  |
|  |  |  | T3 | 1.90  (2.01) | 72 | 2.05 (2.34) | 66 | Mean difference* | 0.150 | 95% CI:  -0.5826 to 0.8826,  p= 0.6862 |  |

N= number of patients analyzed; I= intervention arm; C= control arm; SD= standard deviation; *= self-calculated; T= time point (was not further defined); CIPN= chemotherapy-induced peripheral neuropathy.

| Adverse events | | | | | | | | | | | |
| --- | --- | --- | --- | --- | --- | --- | --- | --- | --- | --- | --- |
| Study | **Definition of outcome** | **Outcome measurement** | **Follow-up** | **I**  **(Event)** | **I**  **(N)** | **C**  **(Event)** | **C**  **(N)** | **Effect measure** | **between-group difference** | **Measure of precision for between group difference** | **Risk of bias** |
| Gilbert 2021 | Patients with at least one AE | Chart review data | NR | 44 (57.9%) | 74 | 31  (41.9 %) | 73 | RR* | 1.4 | 95% CI:  1.01 to 1.941,  p= 0.05 | High |
|  | Patients with at least one postsurgical complication | Chart review data | 30 days | 28 (38.9%) | 74 | 10 (13.7%) | 73 | RR* | 2.76 | 95% CI:  1.448 to 5.267,  p= 0.002 |  |
| Mohile 2021 | Prevalence of any grade 3–5 Common Terminology Criteria for Adverse Events toxic effects (any toxicity) | Chart review data | 3 months | 177 (51%) | 349 | 263 (71%) | 369 | Adjusted RR | 0.74 | 95% CI:  0·64 to 0·86,  p=0·0001 | High |

**eTable 3. Adverse events**

N= number of patients analyzed; I= intervention arm; C= control arm; RR= Risk ratio; *= self-calculated

**eTable 4. Screening**

| Screening | | | | | | | | | | | |
| --- | --- | --- | --- | --- | --- | --- | --- | --- | --- | --- | --- |
| Study | **Definition of outcome** | **Outcome measurement** | **Follow-up** | **I**  **(Event)** | **I**  **(N)** | **C**  **(Event)** | **C**  **(N)** | **Effect measure** | **between-group difference** | **Measure of precision for between group difference** | **Risk of bias** |
| McCarter 2018 | Depression screening** | Chart review data | at Week 1 of RT | 127  (81.3 %) | 156 | 10  (0.7 %) | 151 | OR | 348.82 | 95% CI:  69.31 to 1755.62,  p < 0.0001 | High |
| Gilbert 2021 | Appropriate nutritional assessment | Chart review data | NR | 59 (80%) | 74 | 11 (15%) | 73 | RR* | 5.29 | 95% CI:  3.03 to 9.23,  p <0.0001 | High |

N= number of patients analyzed; I= intervention arm; C= control arm; RT= radiotherapy; OR= Odds ratio; *= self-calculated; **= higher screening rates are better according to recommendation.

**eTable 5. Referral**

| Referral | | | | | | | | | | | |
| --- | --- | --- | --- | --- | --- | --- | --- | --- | --- | --- | --- |
| Study | **Definition of**  **outcome** | **Outcome measurement** | **Follow-up** | **I**  **(Event)** | **I**  **(N)** | **C**  **(Event)** | **C**  **(N)** | **Effect measure** | **between-group difference** | **Measure of precision for between group difference** | **Risk of bias** |
| Brown 2018 | Patient referral to a radiation oncologist | Medical audit of hospital records | within 4 months after RP | 130  (32%) | 407 | 154  (30%) | 505 | RR* | 1.0474 | 95% CI:  0.8631 to 1.2711,  p= 0.6389 | High |
| McCarter 2018 | Depression referral | Chart review data | at Week 1 of RT | 66  (42.1%) | 156 | 0  (0.0%) | 151 | OR | 37.70 | 95% CI:  0.93 to 1530,  p= 0.0537 | High |

N= number of patients analyzed; I= intervention arm; C= control arm; RT= radiotherapy; RR= risk ratio; OR= Odds ratio; *= unadjusted RR, self-calculated.

**eTable 6. Prescribing behaviour**

| Prescribing behaviour | | | | | | | | | | | |
| --- | --- | --- | --- | --- | --- | --- | --- | --- | --- | --- | --- |
| Study | **Definition of**  **outcome** | **Outcome measurement** | **Follow-up** | **I**  **(Result)** | **I**  **(N)** | **C**  **(Result)** | **C**  **(N)** | **Effect measure** | **between-group difference** | **Measure of precision for between group difference** | **Risk of bias** |
| Bonkowski 2018 | Narcotic administrations  24 hours before discharge (less medication being better)  - One dose | Patient chart review (*objective*) | 3 months after inter-vention | 3/44 | 44 | 6/44 | 44 | OR* | 0.4634 | 95% CI:  0.1082 to 1.9845,  p= 0.3000 | Serious |
|  | -Three doses |  |  | 1/44 | 44 | 2/44 | 44 | OR* | 0.4884 | 95% CI:  0.0427 to 5.5912,  p= 0.5645 |  |
|  | Changes in Nursing pain management  practice | Survey- items with 5-point Lickert Scale rating: 1= never,  5= always  (*subjective*)- Item 17 (higher scores are better) | six weeks after inter-vention | Mean 4.00  (SD 0.66) | 25 | Mean 4.00  (SD 0.51) | 25 | Mean difference* | 0.000 | 95% CI:  -0.3354 to 0.3354,  p= 1.000 |  |
|  |  | Item 18 (higher scores are better) |  | Mean 4.20  (SD 0.58) | 25 | Mean 3.76  (SD 0.66) | 25 | Mean difference* | -0.440 | 95% CI:  -0.0867 to 0.7933,  p= 0.0157 |  |
| Knoerl 2021 | Frequency of appropriate CIPN management | Medical records review | NR | 29 (55.77%) | 52 | 29  (63%) | 46 | OR* | 0.7391 | 95% CI:  0.3284 to 1.6634,  p= 0.4651 | Serious |
|  | Frequency of appropriate mild CIPN management |  | NR | 21  (70%) | 30 | 12  (48%) | 25 | OR* | 2.5278 | 95% CI:  0.8356 to 7.6471,  p= 0.1006 |  |
|  | Frequency of appropriate moderate-severe CIPN management |  | NR | 8 (36.36%) | 22 | 8  (40%) | 20 | OR* | 0.8571 | 95% CI:  0.2463 to 2.9827,  p= 0.8086 |  |
| Mohile 2021 | Number of overall medications discontinued before starting cancer treatment regimen | Chart review | At 3 months | NR | 349 | NR | 369 | Mean difference | 0.14 | 95% CI:  0.03 to 0.25,  p= 0.015 | High |
|  | Reduced dose intensity at cycle 1 | Chart review | At 1 cycle | 170 (49%) | 349 | 129 (35%) | 369 | Adjusted RR | 1.38 | 95% CI:  1·06 to 1·78,  p= 0·015 |  |
|  | Dose modification at 3 months | Chart review | At 3 months | 149 (43%) | 349 | 213 (58%) | 369 | Adjusted RR | 0.85 | 95% CI:  0·68 to 1·08,  p= 0·18 |  |
|  | Relative dose intensity | Chart review | At 3 months | 0.63 | 310 | 0.68 | 331 | Mean difference | -0.05 | 95% CI:  –0·09 to –0·01,  p= 0·025 |  |

N= number of participating providers/ patients analyzed; I= intervention arm; C= control arm; SD= standard deviation; *= self-calculated; Item 17: I include non-pharmacologic interventions in my care of patients in pain; Item 18: I make certain that patients who are taking opioids are on a bowel regimen.

**eTable 7. Attitudes**

| Attitudes | | | | | | | | | | | |
| --- | --- | --- | --- | --- | --- | --- | --- | --- | --- | --- | --- |
| Study | **Definition of**  **outcome** | **Outcome measurement** | **Follow-up** | **I**  **(Result)** | **I**  **(N)** | **C**  **(Result)** | **C**  **(N)** | **Effect measure** | **between-group difference** | **Measure of precision for between group difference** | **Risk of bias** |
| Bonkowski 2018 | Changes in attitudes  after intervention | Survey with 5-point Lickert Scale rating: 1= strongly disagree, 5= strongly agree-  *Item 13* (lower scores are better) | six weeks after intervention | Mean 1.84  (SD 0.62) | 25 | Mean 1.64 (SD 0.86) | 25 | Mean difference* | -0.200 | 95% CI:  -0.6263 to 0.2263,  p= 0.3503 | Serious |
|  |  | *Item 14* (lower scores are better) |  | Mean 1.76  (SD 0.52) | 25 | Mean 2.00 (SD 0.91) | 25 | Mean difference* | 0.240 | 95% CI: -0.1815 to 0.6615,  p= 0.2579 |  |
|  |  | Satisfaction Survey  with 5-point Lickert Scale rating: 1= strongly disagree,  5= strongly agree | six weeks after intervention | - highly satisfied with the intervention  - less satisfied with patient-care needs met in the guideline | NR | NR | NR | NR | NR | NR |  |
| Brown 2018 | Attitudes AND  knowledge | Survey scores on a 5-point Likert with scoring 1=Strongly disagree, 2=Disagree, 3=Neither agree nor disagree, 4=Agree, 5=Strongly agree; and “Don’t know” (higher scores are better) | NR | Mean 3.3  (SD 0.7) | 24 | Mean 3.4  (SD 0.5) | 29 | Mean difference | -0.1 | 95% CI:  -0.3 to 0.1,  p= 0.490 | High |
| Cowperth-waite  2019 | Attitudes AND  knowledge | Mean number of correctly answered questions in the KAP survey with 39 total questions | NR | Mean 30.9  (SD 2.42) | 9 | Mean 30.6 (SD 2.46) | 11 | Mean difference* | -0.300 | 95% CI  -2.6063 to 2.0063,  p= 0.7877 | Serious |
| Knoerl 2021 | Attitudes as  Clinician-related acceptability and satisfaction with intervention use and feasibility | Acceptability E - Scale with 8 Items (items were scored from 1 to 5, with higher scores indicating greater acceptability or satisfaction)-  Items 1-8 | after 1 year (at the end of the study) | Mean 3.05  (SD 1.39) | 19 | NR | NR | NR | NR | NR | Serious |
|  |  |  |  | Mean 2.89 (SD.29) | 19 | NR | NR | NR | NR | NR |  |
|  |  |  |  | Mean 3.21  (SD 1.4) | 19 | NR | NR | NR | NR | NR |  |
|  |  |  |  | Mean 3.11 (SD1.15) | 19 | NR | NR | NR | NR | NR |  |
|  |  |  |  | Mean 2.95  (SD 1.13) | 19 | NR | NR | NR | NR | NR |  |
|  |  |  |  | Mean 3.95  (SD 1.13) | 19 | NR | NR | NR | NR | NR |  |
|  |  |  |  | Mean 3.39  (SD 1.14) | 18 | NR | NR | NR | NR | NR |  |
|  |  |  |  | Mean 3.11  (SD 0.94) | 19 | NR | NR | NR | NR | NR |  |
|  |  | Feasibility  Questionnaire with 2 items (items were scored from 1 to 5 (i.e., 1 = Never, 2 = Seldom, 3 = Sometimes, 4 = Frequently, 5 = Always)- Items 9 & 10 |  | Mean 2.58  (SD 0.9) | 19 | NR | NR | NR | NR | NR |  |
|  |  |  |  | Mean 1.89  (SD 0.81) | 19 | NR | NR | NR | NR | NR |  |
| McCarter 2018 | Dietician attitudes towards helpfulness of the practice change strategies | Patient  Health Questionnaire-2  (PHQ-2) | NR | - the majority of dieticians indicated the intervention as helpful/ very helpful | 8 | NR | NR | NR | NR | NR | High |
| Phillips 2017 | Confidence | Self-Perceived Pain Assessment Competencies;  An 11-point visual analogue rating scale ranging  from ‘‘no knowledge/not confident’’ (0) to ‘‘extensive  knowledge and extremely confident’’ (10) was used | directly after intervention | Mean 5.9  (SE 0.58) | 48 | Mean 7.8 (SE 0.28) | 43 | Mean difference | 1.9 | 95% CI:  0.5 to 3.4,  p= 0.012 | Critical |
|  |  |  | 10 weeks after intervention | Mean 9.2  (SE 0.23) | 44 | Mean 7.8 (SE 0.28) | 43 | Mean difference | -1.4 | 95% CI:  -1.9 to -1.0,  p< 0.001 |  |
|  |  |  | difference between intervention and 10 weeks after | Mean 9.2  (SE 0.23) | 44 | Mean 5.9 (SE 0.58) | 48 | Mean difference | -3.4 | 95% CI:  -4.8 to -1.9,  p< 0.001 |  |

N= number of participating providers analyzed; I= intervention arm; C= control arm; SD= standard deviation; SE= standard error; *= self-calculated; **= unclear which outcome measurement (e.g. mean, median) was used, therefore not further calculated. *Item 13:* Pain medicines should be given only when pain is severe*.*; *Item 14*: Patients should experience discomfort prior to receiving the next dose of pain medicine; Item1: Did use of the Algorithm help you identify appropriate areas of concern related to the assessment and/or management of CIPN symptoms?; Item 2: Did use of the Algorithm help guide clinical interactions with patients related to the assessment and management of CIPN symptoms?; Item 3: Was the Algorithm helpful in promoting communication between you and your patients related to the assessment and management of CIPN symptoms?; Item 4: Was the Algorithm helpful in identifying areas of need or symptoms related to CIPN?; Item 5: Was use of the Algorithm helpful in promoting your knowledge related to the assessment and/or management of CIPN symptoms?; Item 6: How understandable was the content presented within the Algorithm?; Item 7: How easy was it to use the Algorithm during your clinical interactions with patients?; Item 8: Overall, how would you rate your satisfaction with the Algorithm?; Item 9: When you received the Chemotherapy-Induced Peripheral Neuropathy (CIPN) Symptom Assessment Summary (sheet displaying patients’ CIPN severity scores) and the CIPN Assessment and Management Algorithm, how often did you use the CIPN Symptom Assessment Summary or Assessment and Management Algorithm to aid you in the assessment and management of CIPN during those particular clinic visits?; Item 10: We sent you several other education materials (i.e., neuropathy safety information, vibration sensibility and deep-tendon reflexes training video, CIPN clinical practice guideline) via email before you began using the CIPN Assessment and Management Algorithm. You most likely received this email around February or March 2019. From the time that you received the email containing the materials, did you review or use the materials we provided to you to aid in the assessment and/or management of CIPN symptoms?

**eTable 8. Knowledge**

| Knowledge | | | | | | | | | | | |
| --- | --- | --- | --- | --- | --- | --- | --- | --- | --- | --- | --- |
| Study | **Definition of**  **outcome** | **Outcome measurement** | **Follow-up** | **I**  **Mean**  **(SD/SE)** | **I**  **(N)** | **C**  **Mean**  **(SD/SE)** | **C**  **(N)** | **Effect measure** | **between-group difference** | **Measure of precision for between group difference** | **Risk of bias** |
| Brown 2018 | Attitudes AND  knowledge | Survey scores on a 5-point Likert** | NR | 3.3  (SD 0.7) | 24 | 3.4  (SD 0.5) | 29 | Mean difference | -0.1 | 95% CI:  -0.3 - 0.1,  p= 0.490 | High |
| Cowperth-waite  2019 | Attitudes AND  knowledge | Mean number of correctly answered questions in the KAP survey with 39 total questions | NR | 30.9  (SD 2.42) | 9 | 30.6  (SD 2.46) | 11 | Mean difference* | -0.300 | 95% CI  -2.6063 to 2.0063,  p= 0.7877 | Serious |
| Phillips 2017 | Perceived knowledge (subjective) | Self-Perceived Pain Assessment Competencies An 11-point visual analogue rating scale ranging  from ‘‘no knowledge/not confident’’ (0) to ‘‘extensive  knowledge and extremely confident’’ (10) was used | directly after intervention | 8.5  (SE 0.33) | 48 | 7.1  (SE 0.31) | 43 | Mean difference | -1.3 | 95% CI:  -2.1 to 0.6,  p< 0.001 | Critical |
|  |  |  | 10 weeks after intervention | 8.9  (SE 2.3) | 44 | 7.1  (SE 0.31) | 43 | Mean difference | -1.7 | 95% CI:  -2.2 to 1.1,  p< 0.001 |  |
|  |  |  | difference between intervention and 10 weeks after | 8.9  (SE 2.3) | 44 | 8.5  (SE 0.33) | 48 | Mean difference | -0.3 | 95% CI:  -1.1 to 0.4,  NS |  |
|  | Assessment tool (subjective) |  | directly after intervention | 6.7  (SE 0.52) | 48 | 3.1  (SE 0.42) | 43 | Mean difference | -3.6 | 95% CI:  -0.5 to 2.2,  p< 0.001 |  |
|  |  |  | 10 weeks after intervention | 6.7  (SE 0.52) | 44 | 3.1  (SE 0.42) | 43 | Mean difference | -3.6 | 95% CI:  -0.5 to 2.2,  p< 0.001 |  |
|  |  |  | difference between intervention and 10 weeks after | 6.7  (SE 0.52) | 44 | 6.7  (SE 0.52) | 48 | Mean difference | 0.0 | 95% CI:  -0.7 to 0.7,  NS |  |

N= number of participating providers analyzed; I= intervention arm; C= control arm; SD= standard deviation; SE= standard error; NS= not significant; *= self-calculated; **= 1= Strongly disagree, 2=Disagree, 3=Neither agree nor disagree, 4=Agree, 5=Strongly agree; and “Don’t know” (higher scores are better).

## **Additional File 12. PRISMA Checklist**

| **Section and Topic** | **Item #** | **Checklist item** | **Location where item is reported** |
| --- | --- | --- | --- |
| **TITLE** | | |  |
| Title | 1 | Identify the report as a systematic review. | Page 1 |
| **ABSTRACT** | | |  |
| Abstract | 2 | See the PRISMA 2020 for Abstracts checklist. | Page 3 & 4 |
| **INTRODUCTION** | | |  |
| Rationale | 3 | Describe the rationale for the review in the context of existing knowledge. | Page 5-7 |
| Objectives | 4 | Provide an explicit statement of the objective(s) or question(s) the review addresses. | Page 7 |
| **METHODS** | | |  |
| Eligibility criteria | 5 | Specify the inclusion and exclusion criteria for the review and how studies were grouped for the syntheses. | Page 7 & 8 |
| Information sources | 6 | Specify all databases, registers, websites, organisations, reference lists and other sources searched or consulted to identify studies. Specify the date when each source was last searched or consulted. | Page 7 |
| Search strategy | 7 | Present the full search strategies for all databases, registers and websites, including any filters and limits used. | Page 7, Figure 1, Additional File 1 |
| Selection process | 8 | Specify the methods used to decide whether a study met the inclusion criteria of the review, including how many reviewers screened each record and each report retrieved, whether they worked independently, and if applicable, details of automation tools used in the process. | Page 7 |
| Data collection process | 9 | Specify the methods used to collect data from reports, including how many reviewers collected data from each report, whether they worked independently, any processes for obtaining or confirming data from study investigators, and if applicable, details of automation tools used in the process. | Page 7 |
| Data items | 10a | List and define all outcomes for which data were sought. Specify whether all results that were compatible with each outcome domain in each study were sought (e.g. for all measures, time points, analyses), and if not, the methods used to decide which results to collect. | Page 7, Table 2, Additional Files |
|  | 10b | List and define all other variables for which data were sought (e.g. participant and intervention characteristics, funding sources). Describe any assumptions made about any missing or unclear information. | Additional Files |
| Study risk of bias assessment | 11 | Specify the methods used to assess risk of bias in the included studies, including details of the tool(s) used, how many reviewers assessed each study and whether they worked independently, and if applicable, details of automation tools used in the process. | Additional Files |
| Effect measures | 12 | Specify for each outcome the effect measure(s) (e.g. risk ratio, mean difference) used in the synthesis or presentation of results. | Page 7 & 8 |
| Synthesis methods | 13a | Describe the processes used to decide which studies were eligible for each synthesis (e.g. tabulating the study intervention characteristics and comparing against the planned groups for each synthesis (item #5)). | Page 7 & 8 |
|  | 13b | Describe any methods required to prepare the data for presentation or synthesis, such as handling of missing summary statistics, or data conversions. | Page 7 & 8 |
|  | 13c | Describe any methods used to tabulate or visually display results of individual studies and syntheses. | Page 7 & 8 |
|  | 13d | Describe any methods used to synthesize results and provide a rationale for the choice(s). If meta-analysis was performed, describe the model(s), method(s) to identify the presence and extent of statistical heterogeneity, and software package(s) used. | Page 7 & 8 |
|  | 13e | Describe any methods used to explore possible causes of heterogeneity among study results (e.g. subgroup analysis, meta-regression). | Page 7 & 8 |
|  | 13f | Describe any sensitivity analyses conducted to assess robustness of the synthesized results. | Page 7 & 8 |
| Reporting bias assessment | 14 | Describe any methods used to assess risk of bias due to missing results in a synthesis (arising from reporting biases). | Page 7 & 8 |
| Certainty assessment | 15 | Describe any methods used to assess certainty (or confidence) in the body of evidence for an outcome. | Page 7 & 8 |
| **RESULTS** | | |  |
| Study selection | 16a | Describe the results of the search and selection process, from the number of records identified in the search to the number of studies included in the review, ideally using a flow diagram. | Page 8, Figure 1 |
|  | 16b | Cite studies that might appear to meet the inclusion criteria, but which were excluded, and explain why they were excluded. | Additional File 2 |
| Study characteristics | 17 | Cite each included study and present its characteristics. | Page 8, Table 2, Additional Files |
| Risk of bias in studies | 18 | Present assessments of risk of bias for each included study. | Page 8 & 9, Additional Files |
| Results of individual studies | 19 | For all outcomes, present, for each study: (a) summary statistics for each group (where appropriate) and (b) an effect estimate and its precision (e.g. confidence/credible interval), ideally using structured tables or plots. | Page 9-12, eTables 1-8 (Additional Files) |
| Results of syntheses | 20a | For each synthesis, briefly summarise the characteristics and risk of bias among contributing studies. | Page 9-13 |
|  | 20b | Present results of all statistical syntheses conducted. If meta-analysis was done, present for each the summary estimate and its precision (e.g. confidence/credible interval) and measures of statistical heterogeneity. If comparing groups, describe the direction of the effect. | Page 9-13 |
|  | 20c | Present results of all investigations of possible causes of heterogeneity among study results. | Page 9-13 |
|  | 20d | Present results of all sensitivity analyses conducted to assess the robustness of the synthesized results. | Page 9-13 |
| Reporting biases | 21 | Present assessments of risk of bias due to missing results (arising from reporting biases) for each synthesis assessed. | Page 9-13 |
| Certainty of evidence | 22 | Present assessments of certainty (or confidence) in the body of evidence for each outcome assessed. | Page 9-13, Table 3 |
| **DISCUSSION** | | |  |
| Discussion | 23a | Provide a general interpretation of the results in the context of other evidence. | Page 13-14 |
|  | 23b | Discuss any limitations of the evidence included in the review. | Page 14 |
|  | 23c | Discuss any limitations of the review processes used. | Page 14 |
|  | 23d | Discuss implications of the results for practice, policy, and future research. | Page 15-17, Figure 4 |
| **OTHER INFORMATION** | | |  |
| Registration and protocol | 24a | Provide registration information for the review, including register name and registration number, or state that the review was not registered. | Page 18 |
|  | 24b | Indicate where the review protocol can be accessed, or state that a protocol was not prepared. | - |
|  | 24c | Describe and explain any amendments to information provided at registration or in the protocol. | - |
| Support | 25 | Describe sources of financial or non-financial support for the review, and the role of the funders or sponsors in the review. | Page 17 |
| Competing interests | 26 | Declare any competing interests of review authors. | Page 17 |
| Availability of data, code and other materials | 27 | Report which of the following are publicly available and where they can be found: template data collection forms; data extracted from included studies; data used for all analyses; analytic code; any other materials used in the review. | - |

*From:*  Page MJ, McKenzie JE, Bossuyt PM, Boutron I, Hoffmann TC, Mulrow CD, et al. The PRISMA 2020 statement: an updated guideline for reporting systematic reviews. BMJ 2021;372:n71. doi: 10.1136/bmj.n71.

## **References**

1. Brown B, Young J, Smith DP, Kneebone AB, Brooks AJ, Egger S, et al. A multidisciplinary team-oriented intervention to increase guideline recommended care for high-risk prostate cancer: A stepped-wedge cluster randomised implementation trial. Implementation science : IS. 2018;13(1):43.

2. Saulle R, Sinopoli A, De Paula Baer A, Mannocci A, Marino M, De Belvis AG, et al. The PRECEDE-PROCEED model as a tool in Public Health screening: a systematic review. La Clinica terapeutica. 2020;171(2):e167-e77.

3. EPOC Taxonomy 2015. Available from: <https://zenodo.org/record/5105851#.Yue9J2PP1PY> (last accessed: 20.01.2023).

4. Gilbert T, Bernard L, Alex, re M, Bin-Dorel S, Villeneuve L, et al. Impact of a Geriatric Intervention to Improve Screening and Management of Undernutrition in Older Patients Undergoing Surgery for Colorectal Cancer: Results of the ANC Stepped-Wedge Trial. 2021;13(7).

5. Lovell MR, Phillips JL, Luckett T, Lam L, Boyle FM, Davidson PM, et al. Effect of Cancer Pain Guideline Implementation on Pain Outcomes Among Adult Outpatients With Cancer-Related Pain: a Stepped Wedge Cluster Randomized Trial. 2022;5(2):e220060.

6. Michie S, van Stralen MM, West R. The behaviour change wheel: a new method for characterising and designing behaviour change interventions. Implementation science : IS. 2011;6:42.

7. McCarter K, Baker AL, Britton B, Beck AK, Carter G, Bauer J, et al. Effectiveness of clinical practice change strategies in improving dietitian care for head and neck cancer patients according to evidence-based clinical guidelines: a stepped-wedge, randomized controlled trial. Translational behavioral medicine. 2018;8(2):166-74.

8. Mohile SG, Mohamed MR, Xu H, Culakova E, Loh KP, Magnuson A, et al. Evaluation of geriatric assessment and management on the toxic effects of cancer treatment (GAP70+): a cluster-randomised study. 2021;398(10314):1894-904.

9. Bonkowski SL, De Gagne JC, Cade MB, Bulla SA. Evaluation of a Pain Management Education Program and Operational Guideline on Nursing Practice, Attitudes, and Pain Management. Journal of continuing education in nursing. 2018;49(4):178-85.

10. Field B, Booth A, Ilott I, Gerrish K. Using the Knowledge to Action Framework in practice: a citation analysis and systematic review. Implementation Science. 2014;9(1):1-14.

11. Cowperthwaite SM, Kozachik SL. Improving the Pain Experience for Hospitalized Patients With Cancer. Oncology nursing forum. 2019;46(2):198-207.

12. Campbell B. Applying knowledge to generate action: A community‐based knowledge translation framework. Journal of Continuing Education in the Health Professions. 2010;30(1):65-71.

13. Knoerl R, Mazzola E, Hong F, Salehi E, McCleary N, Ligibel J, et al. Exploring the impact of a decision support algorithm to improve clinicians' chemotherapy-induced peripheral neuropathy assessment and management practices: a two-phase, longitudinal study. BMC cancer. 2021;21(1):236.

14. Phillips JL, Heneka N, Hickman L, Lam L, Shaw T. Can A Complex Online Intervention Improve Cancer Nurses' Pain Screening and Assessment Practices? Results from a Multicenter, Pre-post Test Pilot Study. Pain management nursing : official journal of the American Society of Pain Management Nurses. 2017;18(2):75-89.

15. Michie S, Van Stralen MM, West R. The behaviour change wheel: a new method for characterising and designing behaviour change interventions. Implementation science. 2011;6(1):1-12.
